# Synthesis and evaluation of 3-alkynyl-5-aryl-7-aza-indoles as broad-spectrum antiviral agents

**DOI:** 10.3389/fchem.2022.1058229

**Published:** 2022-10-26

**Authors:** Belén Martinez-Gualda, Mirthe Graus, Anita Camps, Emiel Vanhulle, Sirle Saul, Siavash Azari, Do Hoang Nhu Tran, Laura Vangeel, Winston Chiu, Johan Neyts, Dominique Schols, Shirit Einav, Kurt Vermeire, Steven De Jonghe

**Affiliations:** ^1^ KU Leuven, Department of Pharmaceutical and Pharmacological Sciences, Rega Institute for Medical Research, Laboratory of Medicinal Chemistry, Leuven, Belgium; ^2^ KU Leuven, Department of Microbiology, Immunology and Transplantation, Rega Institute for Medical Research, Laboratory of Virology and Chemotherapy, Leuven, Belgium; ^3^ Department of Medicine, Division of Infectious Diseases and Geographic Medicine, Stanford University, Stanford, CA, United States; ^4^ Department of Microbiology and Immunology, Stanford University, Stanford, CA, United States; ^5^ Chan Zuckerberg Biohub, San Francisco, CA, United States

**Keywords:** 7-aza-indole, pyrrolo[2,3-b]pyridine, respiratory syncytial virus (RSV), severe acute respiratory syndrome coronavirus 2 (SARS-CoV-2), Venezuelan equine encephalitis virus (VEEV), adaptor-associated kinase 1

## Abstract

RNA viral infections, including those caused by respiratory syncytial virus (RSV), severe acute respiratory syndrome coronavirus 2 (SARS-CoV-2), and Venezuelan Equine encephalitis virus (VEEV), pose a major global health challenge. Here, we report the synthesis and screening of a series of pyrrolo[2,3-*b*]pyridines targeting RSV, SARS-CoV-2 and/or VEEV. From this campaign, a series of lead compounds was generated that demonstrated antiviral activity in the low single-digit micromolar range against the various viruses and did not show cytotoxicity. These findings highlight the potential of 3-alkynyl-5-aryl-7-aza-indoles as a promising chemotype for the development of broad-spectrum antiviral agents.

## 1 Introduction

Viruses causing respiratory and brain infections are major causes of morbidity and mortality in humans. Examples of clinically relevant prevalent respiratory viruses include the influenza virus, rhinovirus, metapneumovirus, parainfluenza virus, respiratory syncytial virus and various coronaviruses (Nichols et al., 2008; Leung, 2021). Viruses from the alphavirus, flavivirus, and herpesvirus families are among the multiple viral pathogens implicated in encephalitis (Swanson and McGavern, 2015).

The human respiratory syncytial virus (RSV) is a negative sense, single-strand RNA virus that belongs to the Pneumoviridae family and is classified within the Orthopneumovirus genus (Griffiths et al., 2017). Worldwide, RSV is the most important etiological agent of acute lower respiratory tract infections in infants and young children. RSV infects more than 70% of infants before their first year of life, and nearly every child by the age of 2. RSV infection of the lower respiratory tract results in airway inflammation, bronchiolitis, pneumonia, coughing, wheezing and in some cases, respiratory failure (Piedimonte and Perez, 2014). Beyond being an important pathogen for infants, RSV can also produce severe complications in the elderly and high-risk adults (such as immunosuppressed post-transplant patients) and it is the second most frequent cause of “excess deaths” during the winter months in this population, after influenza virus infections (Falsey et al., 2005).

Despite the great clinical impact of RSV, there are currently no approved vaccines or therapeutic antiviral drugs against RSV (Costello et al., 2012), and the standard of care for the management of RSV infection is limited to supportive care, including the administration of fluids and oxygen. Immunoprophylaxis with palivizumab, an RSV-specific humanized monoclonal antibody, is recommended only for the prevention of RSV infection in infants who are born prematurely or who are at high risk. However, it has been reported that Palivizumab, has little therapeutic benefit. Ribivarin, a nucleoside analogue, is the only small molecule drug that received marketing approval for the treatment of RSV infection, but it has a limited utility due to its controversial efficacy, toxicity concerns and an inconvenient route of inhaled administration.

**FIGURE 1:**
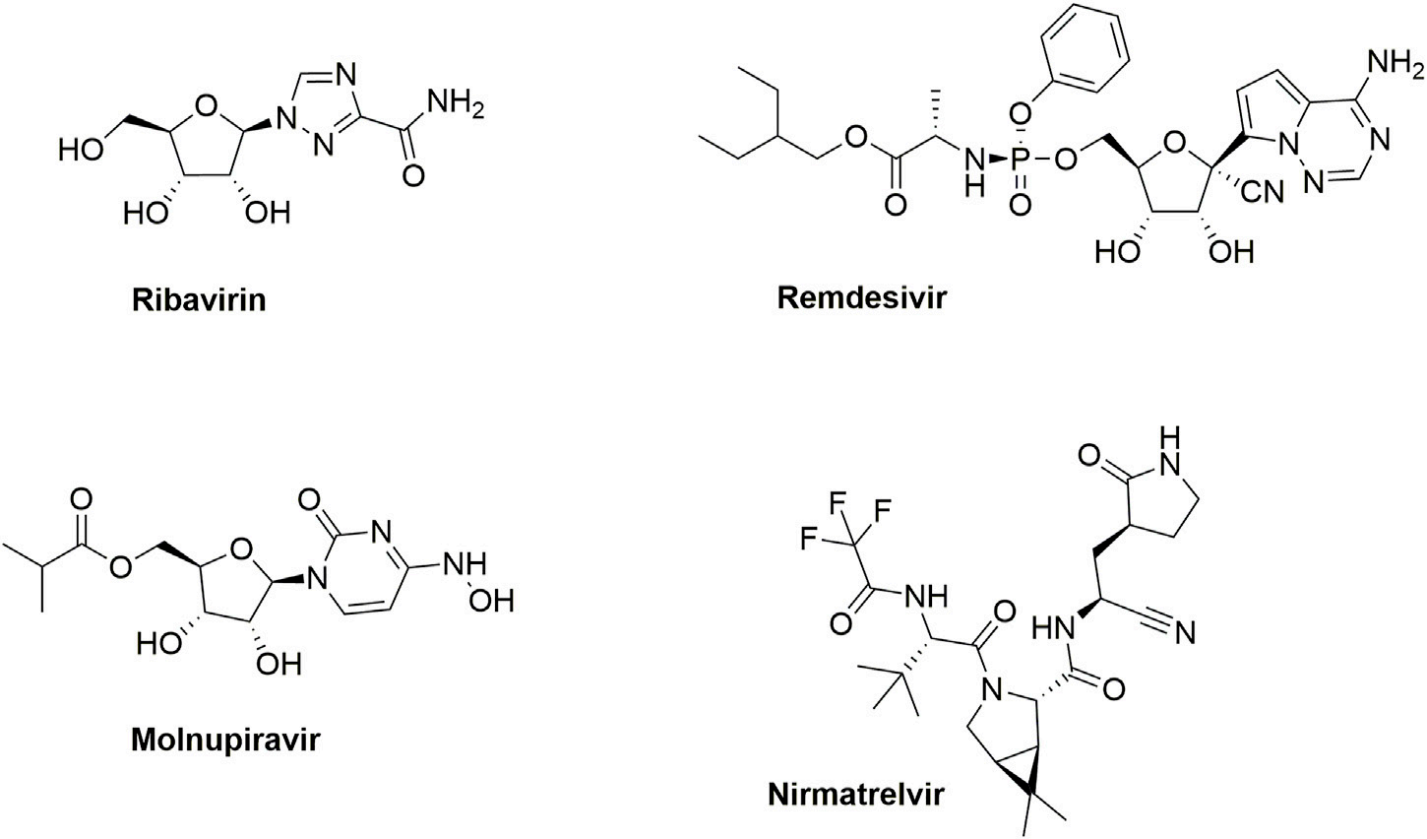
Marketed drugs for RSV and SARS-CoV-2 infections.

Another important respiratory virus is the severe acute respiratory syndrome coronavirus 2 (SARS-CoV-2), which is a betacoronavirus that belongs to the family of Coronaviridae, and the subfamily of Orthocoronavirinae (V’kovski et al., 2021). Infection with SARS-CoV-2 results in viral sepsis, pneumonia and hypoxemic respiratory failure, collectively known as coronavirus disease 2019 (COVID-19). SARS-CoV-2 emerged in December 2019 in Wuhan (China) and was declared as a pandemic by the World Health Organization in March 2020. It spurred the development of various COVID-19 vaccines, employing a broad range of technology platforms (Zhang et al., 2022). In addition, various small molecule therapeutics were discovered (Figure 1) (Ho et al., 2022). Remdesivir is a phosphoramidate prodrug of the *C*-nucleoside GS-441524 that received FDA approval for the treatment of adult and pediatric (aged >12 years) SARS-CoV-2-infected patients. Since it requires intravenous administration, remdesivir usage is limited to hospital settings. In contrast, molnupiravir (another RNA-dependent RNA polymerase inhibitor) is orally bioavailable. Nirmatrelvir is a peptidomimetic targeting the main protease (Mpro or 3CLpro) of SARS-CoV-2. Nirmatrelvir and molnupiravir both received marketing approval in many countries for the treatment of mild-to-moderate COVID-19 in non-hospitalised patients who are at high risk of progressing to severe COVID-19.

VEEV is an alphavirus implicated in neurological disease in Central and South America (Sharma and Knollmann-Ritschel, 2019). Most VEEV infections in humans are mild, yet, approximately 14% of the patients develop encephalitis, which could lead to neurological deficits and sometimes death (Aguilar et al., 2020). VEEV is predominantly transmitted *via* a mosquito bite; however, since it can also be transmitted *via* aerosol exposure, it is considered a major bioterrorism threat (Hawley and Eitzen, 2001). Despite the importance of effective countermeasures, there are neither approved antiviral drugs nor licensed human vaccines available for VEEV infection currently.

## 2 Results and discussion

### 2.1 Hit identification

To discover hits that can be used as a starting point for a medicinal chemistry optimization campaign towards novel anti-RSV agents, various in-house available compound libraries were screened for anti-RSV activity in HEp-2 (cervical adenocarcinoma cells) *via* a cytopathic effect (CPE) assay with a colorimetric read-out. In parallel, cytotoxicity was measured in uninfected HEp-2 cells *via* a colorimetric formazan-based MTS cell viability assay, allowing calculation of selectivity indexes (SI). Since RSV-A is more prevalent than the serologically distinct RSV-B (Laham et al., 2017), the primary screening was conducted with RSV-A. Ribavirin (an inosine monophosphate dehydrogenase inhibitor) (De Clercq, 1996), dextran sulfate sodium salt (De Clercq, 1996) (MW 10,000; DS-10000; interferes with the virus adsorption to the cell), and TMC353121 (an RSV fusion inhibitor) (Bonfanti et al., 2008) were included as positive controls, yielding EC_50_ and CC_50_ values that are in agreement with literature data (Table 1).

**TABLE 1:** Anti-RSV activity of reference and hit compounds.

Cmpd#	RSV-A EC_50_ (µM)^a^	HEp-2 CC_50_ (µM)^b^
Ribavirin	3.11 ± 1.85	> 50
DS-10000	0.068 ± 0.045	> 50
TMC353121	0.0013 ± 0.0008	> 10
1	0.19 ± 0.13	2

^a^ 50% effective concentration, or concentration required to inhibit virus-induced cytopathogenicity in HEp-2 cells by 50%.

^b^ 50% cytotoxic concentration. or concentration required to reduce viability of HEp-2 cells by 50%.

**FIGURE 2:**
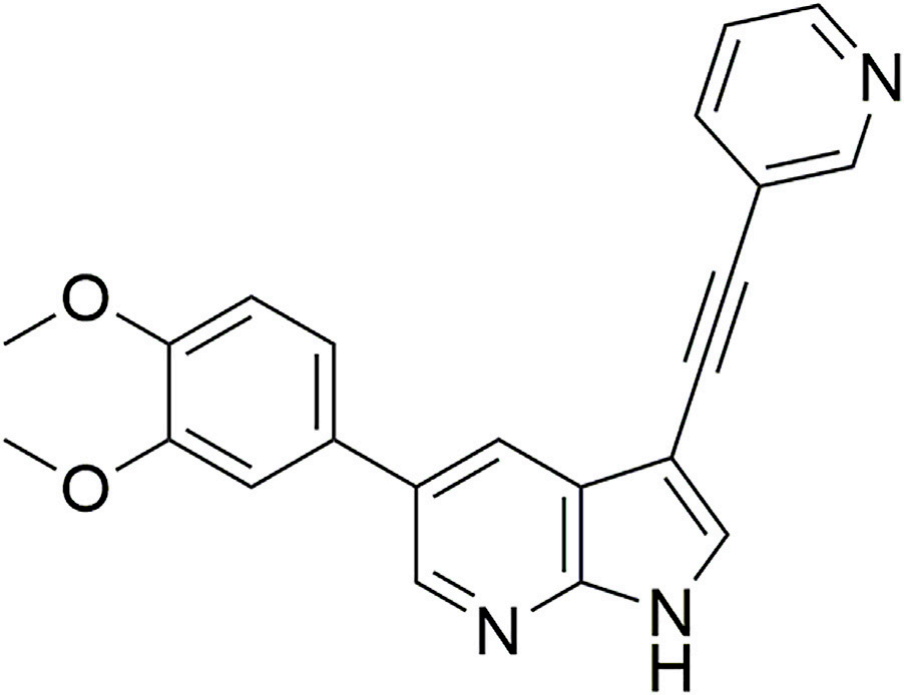
RSV hit compound **1**.

From this screening, compound **1** (Figure 2) emerged showing promising inhibition of RSV-A-induced CPE with an EC_50_ value of 0.19 µM. The cytotoxicity (CC_50_ value) was determined as 2 μM, yielding a rather low selectivity index (SI = 10.5). Compound **1** was originally synthesized as a potent inhibitor of adaptor-associated kinase 1 (AAK1) with an IC_50_ value of 4 nM in an enzymatic assay (Verdonck et al., 2019). AAK1 is a cellular kinase that plays an important role in clathrin-mediated endocytosis. AAK1 regulates hepatitis C virus (HCV) entry and assembly, and also plays a crucial role in the viral life cycle of the dengue virus (DENV) and Ebola virus (EBOV) (Neveu et al., 2012; Bekerman et al., 2017). In agreement with these data, compound **1** was previously shown to display promising antiviral activity against DENV and EBOV (Verdonck et al., 2019). All other AAK1 inhibitors screened here showed no antiviral activity against RSV, suggesting that the anti-RSV activity of compound **1** is independent of AAK1 inhibition. Compound **1** was considered as a promising hit for the discovery of novel antiviral agents based on an unknown mode of action.

### 2.2 Chemistry

**FIGURE 3:**
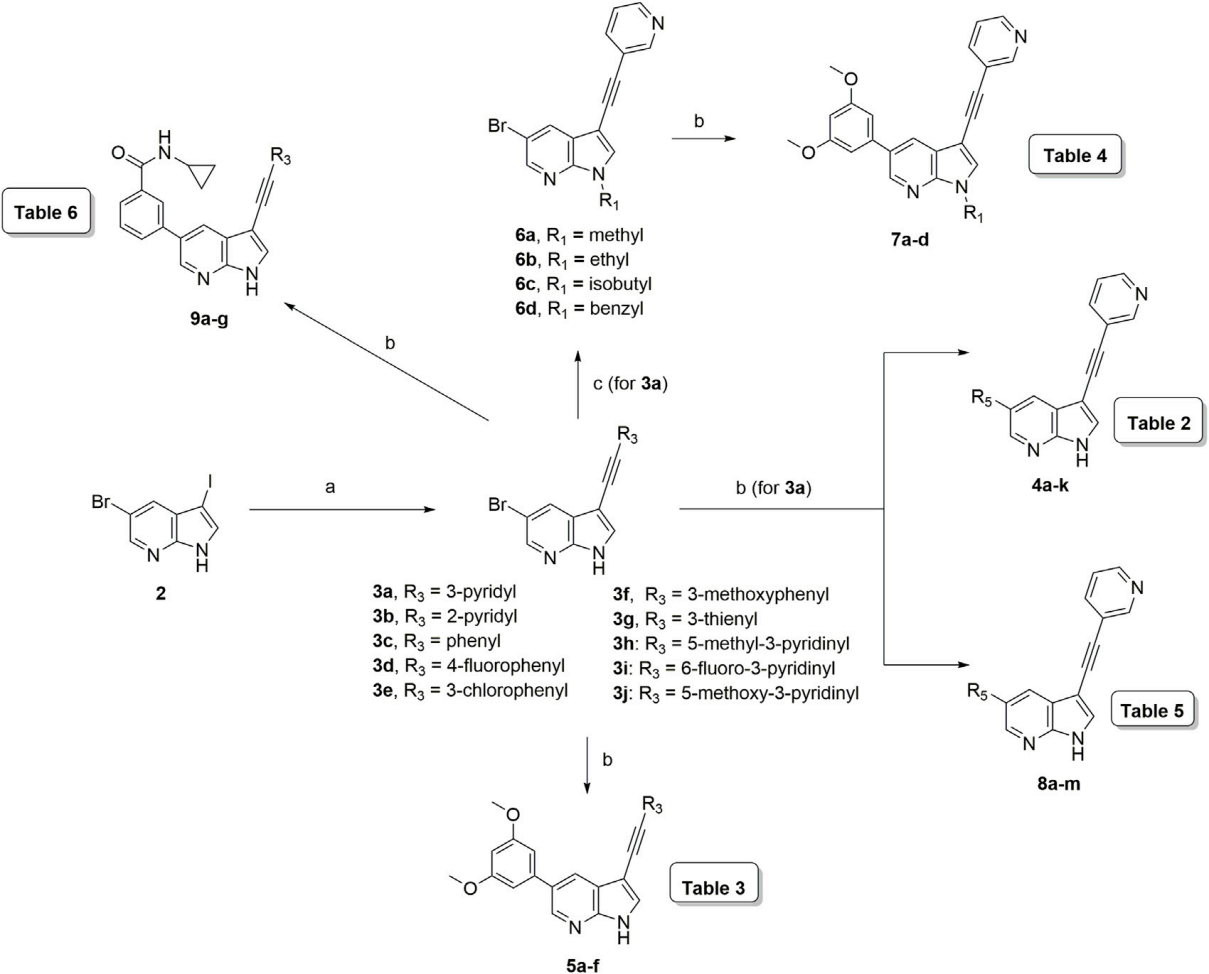
Synthesis scheme of a 3-alkynyl-5-aryl-7-aza-indole library. *Reagents and conditions*. **(A)** R_3_CCH, CuI, Pd(PPh_3_)_2_Cl_2_, Et_3_N, THF, 30°C; **(B)** R_5_B(OH)_2_, Pd(PPh_3_)_4_, K_2_CO_3_, dioxane/water, reflux; **(C)** RI or RBr, NaH, dry THF, 0°C to rt, overnight.

The synthesis started from commercially available 5-bromo-3-iodo-1*H*-pyrrolo[2,3-*b*]pyridine **2** (Figure 3). A series of (hetero)aromatic acetylenes was regioselectively introduced at position 3 of the pyrrolo[2,3-*b*]pyridine scaffold *via* a palladium-catalyzed cross-coupling Sonogashira reaction (Verdonck et al., 2019). Standard reaction conditions were applied, using an appropriate acetylene, copper iodide, Pd(PPh_3_)_2_Cl_2_ as catalyst, triethylamine as base and tetrahydrofuran as solvent. Although the various acetylenes were dropwise to the reaction mixture (as a solution in THF) over a period of 30 min, a substantial amount of a side product was formed and TLC monitoring indicated that the acetylenes were completely consumed before disappearance of the starting material **2**. This was attributed to the copper-catalyzed oxidative homocoupling of the acetylenes, known as the Glaser reaction, yielding diacetylene derivatives (Glaser, 1869; Glaser, 1870). It has been shown that the presence of oxygen or nitrogen ligands, such as tertiary amines, contribute to this side reaction (Valentí et al., 1990; Li et al., 2009; Zheng et al., 2010). To avoid this homocoupling and hence, to increase the yield of the desired 3-alkynyl pyrrolo[2,3-*b*]pyridines, a continuous flow of argon was passed through the reaction mixture prior to the addition of the catalyst and also during the addition of the corresponding acetylene, affording compounds **3a-g** in yields ranging from 24 to 85%. The subsequent Suzuki coupling of 5-bromo-3-(pyridin-3-ylethynyl)-1*H*-pyrrolo[2,3-*b*]pyridine **3a** with an array of arylboronic acids or arylpinacolboronate esters, using Pd(PPh_3_)_4_ as catalyst, potassium carbonate as base in a mixture of dioxane and water, afforded the final compounds **4a-k** and **8a-m**. Similarly, intermediates **3a-g** were subjected to a Suzuki reaction with 3,5-dimethoxyphenylboronic acid and 3-(*N*-cyclopropylaminocarbonyl)phenylboronic acid pinacol ester, affording the final compounds **5a-f** and **9a-g** respectively, in yields varying from 39 to 81%.

The pyrrole nitrogen of intermediate **3a** was used for alkylation reactions with different alkylhalides under basic conditions, furnishing compounds **6a-d** in excellent yields (79–91%). The Suzuki coupling of compounds **6a-d** with 3,5-dimethoxyphenylboronic acid yielded final compounds **7a-d** in moderate yields (72–76%).

## 3 Evaluation of anti-RSV activity

Since a large number of arylboronic acids are commercially available, the SAR exploration started to probe the optimal substitution pattern of the phenyl moiety at position five of the 7-aza-indole scaffold of hit compound **1**. Initially, the focus was on alkoxy- and halogen-substituted phenyl analogues, as well as on the replacement of the phenyl moiety by various heteroaromatics (Table 2). This site of the molecule tolerates quite some structural variety, since only compounds **4f** and **4j**, having a 4-methoxyethoxyphenyl and a furanyl moiety, respectively, are completely devoid of anti-RSV activity. Other derivatives are endowed with good antiviral activity, although several among them (compounds **4a**, **4b**, **4c** and **4e**) showed prominent cytotoxicity in HEp-2 cells. The 3-methoxy-4-pyridyl (compound **4g**), the 3-chlorophenyl (compound **4h**), the 4-fluorophenyl (compound **4i**) and the 3-thienyl (compound **4k**) derivatives have an attractive profile, displaying antiviral activity against RSV in the low µM range with no cytotoxicity. Compound **4d**, bearing a 2,5-dimethoxyphenyl moiety, emerged as the most promising derivative, displaying good antiviral activity (EC_50_ = 0.55 µM) with no cytotoxicity (CC_50_ > 50 µM), affording a selectivity index of 90.

**TABLE 2:** Anti-RSV activity and cytotoxicity of 5-aryl-3-(pyridin-3-yl-ethynyl)-pyrrolo[2,3-*b*]pyridines.

. 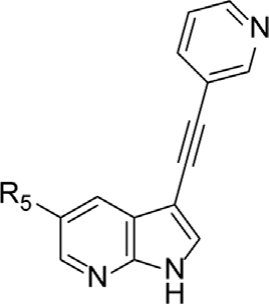			
Cmpd#	R_5_	RSV-A EC_50_ (µM)^a^	HEp-2 CC_50_ (µM)^b^
**1**	. 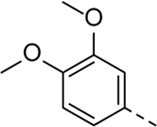	0.19 ± 0.13	2
**4a**	. 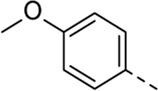	0.048 ± 0.037	0.24 ± 0.23
**4b**	. 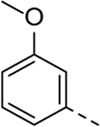	0.67 ± 0.43	6.00 ± 5.66
**4c**	. 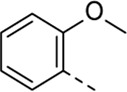	0.69 ± 0.32	6.00 ± 5.66
**4d**	. 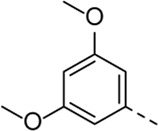	0.55 ± 0.41	>50
**4e**	. 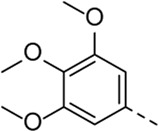	0.71 ± 0.55	1.20 ± 1.13
**4f**	. 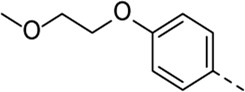	30.00 ± 28.28	> 50
**4g**	. 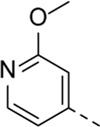	2.71 ± 1.01	> 50
**4h**	. 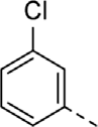	2.89 ± 2.24	> 50
**4i**	. 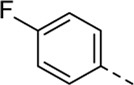	2.23 ± 1.32	> 50
**4j**	. 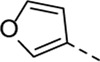	47.83 ± 3.07	> 50
**4k**	. 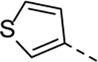	4.83 ± 1.44	> 50

^a^ 50% effective concentration, or concentration required to inhibit virus-induced cytopathogenicity in HEp-2 cells by 50%.

^b^ 50% cytotoxic concentration, or concentration required to reduce viability of HEp-2 cells by 50%.

Since compound **4d** had a potent antiviral activity with no toxicity, the 2,5-dimethoxyphenyl moiety was kept fixed in the synthesis of a new set of analogues, and structural variation was introduced at position three of the pyrrolo[2,3-*b*]pyridine scaffold (Table 3). Unfortunately, none of the structural modifications yielded analogues with an attractive profile. Several congeners (compounds **5a**-**c** and **5f**) were cytotoxic and lacked selective antiviral activity. The 7-aza-indoles with a 3-chloro- (compound **5d**) or 3-methoxy (compound **5e**) phenylacetylene substituent at position three are devoid of cytotoxicity, but showed a decreased activity against RSV-A, relative to compound **4d** (30-fold loss for compound **5d**; 5-fold decreased activity for compound **5e**).

**TABLE 3:** Anti-RSV activity and cytotoxicity of 5-(2,5-dimethoxy)-3-alkynyl-pyrrolo[2,3-*b*]pyridines

. 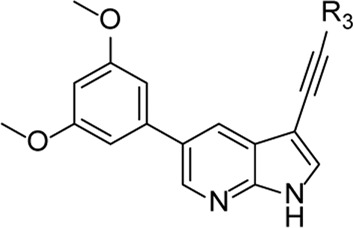			
Cmpd#	R_3_	RSV-A EC_50_ (µM)^a^	HEp-2 CC_50_ (µM)^b^
**4d**	. 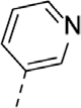	0.55 ± 0.41	> 50
**5a**	. 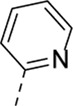	0.08	0.24 ± 0.23
**5b**	. 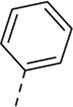	1.59 ± 0.59	6.00 ± 4.35
**5c**	. 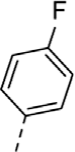	0.36 ± 0.064	1.66 ± 0.89
**5d**	. 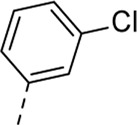	16.30	> 50
**5e**	. 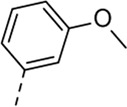	2.75 ± 0.24	> 50
**5f**	. 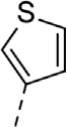	0.31 ± 0.13	1.84 ± 0.95

^a^ 50% effective concentration, or concentration required to inhibit virus-induced cytopathogenicity in HEp-2 cells by 50%.

^b^ 50% cytotoxic concentration, or concentration required to reduce viability of HEp-2 cells by 50%.

To further explore the SAR of compound **4** as an anti-RSV agent, the pyrrole nitrogen was substituted with various alkyl groups (Table 4). The presence of a methyl group (compound **7a**) largely retained antiviral activity, whereas increasing the bulk of the substituent to an ethyl (compound **7b**), isobutyl (compound **7c**) or benzyl (compound **7d**) afforded compounds with reduced antiviral activity or increased toxicity.

**TABLE 4:** Anti-RSV activity and cytotoxicity of N-substituted 5-(2,5-dimethoxy)-3-(3-pyridinyl)-pyrrolo[2,3-*b*]pyridines

. 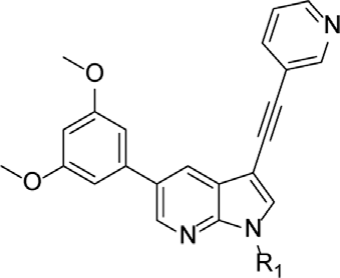			
Cmpd#	R_1_	RSV-A EC_50_ (µM)^a^	HEp-2 CC_50_ (µM)^b^
**7a**	methyl	1.65 ± 0.50	30.00 ± 28.28
**7b**	ethyl	> 2	2.00 ± 0.00
**7c**	isobutyl	> 10	10.70 ± 0.98
**7d**	benzyl	4.88 ± 0.28	> 50

^a^ 50% effective concentration, or concentration required to inhibit virus-induced cytopathogenicity in HEp-2 cells by 50%.

^b^ 50% cytotoxic concentration, or concentration required to reduce viability of HEp-2 cells by 50%.

Structural modifications at positions one and three of the scaffold (Tables 3, 4) had a detrimental effect on antiviral activity. However, preliminary SAR (Table 2) indicated that it was feasible to modulate the antiviral activity and cytotoxicity by structural variation at position 5, and therefore this area was considered attractive for further exploration in another round of SAR. A carboxylic acid on the phenyl moiety can function as a chemical handle for the introduction of a broad structural variety by the subsequent formation of amides. To determine if this is a viable approach, a number of commercially available carboxamide substituted arylboronic acids (either *meta* or *para*-substituted) were coupled with the key intermediate **3a**, yielding compounds **8a-m** (Table 5). Subtle structural modifications within this series profoundly influenced the activity and toxicity. An encouraging level of antiviral potency (EC_50_ of 0.89 µM) was observed with the first analogue within this series, i.e., the *N*-methylbenzamide analogue **8a**. Elongation of the methyl to an ethyl group furnished compound **8b** that has comparable anti-RSV activity to compound **8a**, but less cytotoxicity (CC_50_ values of 6 μM and 50 µM for compounds **8a** and **8b**, respectively). The *N,N*-diethylbenzamide analogue **8c** showed antiviral activity (EC_50_ = 0.47 µM) with only mild cytotoxicity (CC_50_ = 10.94 µM). The *meta*-substituted congener (compound **8e**) was more potent and less cytotoxic relative to the *para*-substituted analogue **8a**. Increasing the steric bulk of the substituent on the amide nitrogen to a cyclopropyl (compound **8f**), an isobutyl (compound **8g**), a methoxyethyl (compound **8h**) or a benzyl (compound **8i**) moiety, was largely well-tolerated, with EC_50_ values between 0.1 and 2.7 µM, although some analogues, such as compounds **8h** (CC_50_ = 17.33 µM) and particularly **8g** (CC_50_ = 3.38 µM), were strikingly more cytotoxic. A series of tertiary amides (compounds **8j**-**m**) was also prepared, from which the *N,N*-dimethylbenzamide derivative **8j** displayed low µM activity and no cytotoxicity.

**TABLE 5:** Anti-RSV activity and cytotoxicity of 5-aryl-3-(3-pyridinyl)pyrrolo[2,3-*b*]pyridines

. 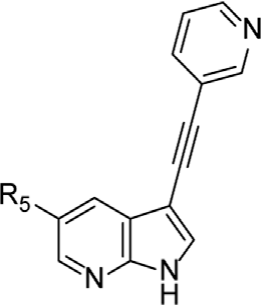			
Cmpd#	R_5_	RSV-A EC_50_ (µM)^a^	HEp-2 CC_50_ (µM)^b^
**8a**	. 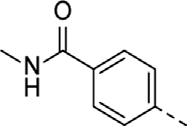	0.89 ± 0.40	6.00 ± 5.66
**8b**	. 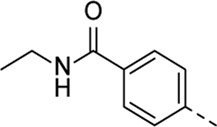	0.30 ± 0.28	50
**8c**	. 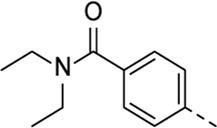	0.47 ± 0.73	10.94
**8d**	. 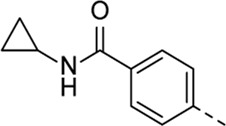	4.63 ± 3.71	50.00 ± 0.0
**8e**	. 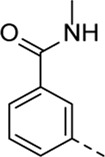	0.29 ± 0.32	> 50
**8f**	. 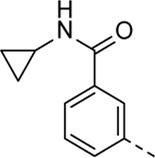	0.16 ± 0.025	> 50
**8g**	. 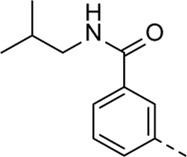	0.66 ± 0.22	3.38 ± 1.01
**8h**	. 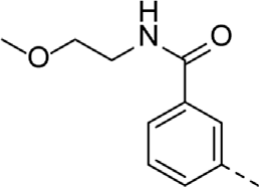	0.72 ± 0.46	17.33 ± 7.88
**8i**	. 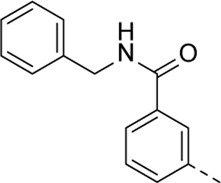	2.71 ± 2.88	> 50
**8j**	. 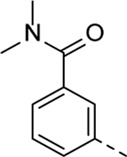	3.65 ± 2.28	> 50
**8k**	. 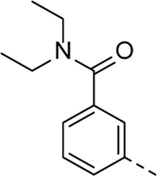	0.97 ± 0.18	4.04 ± 0.14
**8L**	. 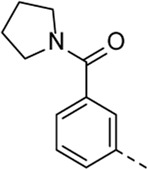	1.00 ± 0.94	5.71 ± 0.044
**8m**	. 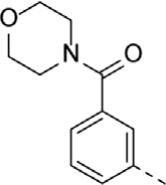	3.42 ± 4.40	10.90 ± 0.69

^a^ 50% effective concentration, or concentration required to inhibit virus-induced cytopathogenicity in HEp-2 cells by 50%.

^b^ 50% cytotoxic concentration, or concentration required to reduce viability of HEp-2 cells by 50%.

Encouraged by the selectivity index of compound **8f**, the *N*-cyclopropyl-carboxamide substituent was retained for further optimisation, and substitution at position 3 of the central core was reinvestigated (Table 6). Substitution of the 3-pyridinyl moiety with a fluorine (compound **9a**) caused a 10-fold loss in activity, whereas the presence of a methoxy (compound **9b**) or a methyl group (compound **9c**) largely retained antiviral activity. Replacement of the pyridinyl moiety of compound **8h** by a phenyl or thienyl ring yielded compounds **9d** and **9e**, respectively, with comparable activity to compound **8h**, indicating that the nitrogen is not essential for activity against RSV. Substitution of the phenyl with a chlorine afforded the inactive analogue **9f**, whereas the corresponding 3-methoxyphenyl derivative **9g** showed activity, suggesting that the electronic nature of the substituents on the phenyl ring has a profound effect on the anti-RSV activity.

**TABLE 6:** Anti-RSV activity and cytotoxicity of 5-(*N*-cyclopropylbenzamide)-3-alkynyl-pyrrolo[2,3-*b*]pyridines

. 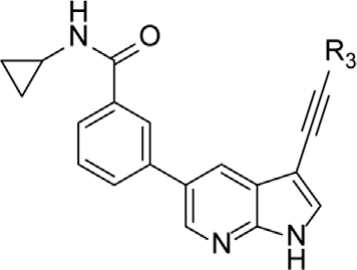			
Cmpd#	R_5_	RSV-A EC_50_ (µM)^a^	HEp-2 CC_50_ (µM)^b^
**8f**	. 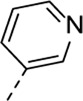	0.16 ± 0.025	> 50
**9a**	. 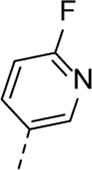	1.76 ± 0.91	> 50
**9b**	. 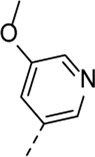	0.24 ± 0.057	22.20 ± 3.68
**9c**	. 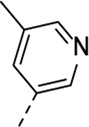	0.18 ± 0.085	> 50
**9d**	. 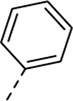	0.82 ± 0.22	> 50
**9e**	. 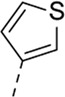	0.51 ± 0.16	> 50
**9f**	. 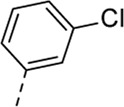	> 50	> 50
**9g**	. 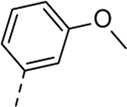	0.10 ± 0.19	> 20

^a^ 50% effective concentration, or concentration required to inhibit virus-induced cytopathogenicity in HEp-2 cells by 50%.

^b^ 50% cytotoxic concentration, or concentration required to reduce viability of HEp-2 cells by 50%.

To exclude a cell-type specific antiviral effect, the anti-RSV-A activity of the most promising compounds was studied in the more biologically relevant A549 cell line (human lung epithelial cells) by measuring the viral RNA levels in the culture supernatant *via* RT-qPCR. Interestingly, compounds **4d** (EC_50_ = 0.05 µM), **8b** (EC_50_ = 0.01 µM), **8e** (EC_50_ = 0.20 µM), and **8f** (EC_50_ = 0.59 µM) showed potent antiviral activity in this orthogonal assay.

Since the initial hit **1** is a potent AAK1 inhibitor, a selection of promising RSV compounds was investigated for AAK1 affinity by the LanthaScreen™ Eu Kinase Binding Assay (ThermoFisher Scientific), in which binding of an Alexa Fluor™ conjugate or “tracer” to a kinase is detected by addition of an Eu-labelled anti-tag antibody (Verdonck et al., 2019). Binding of the tracer and antibody to a kinase results in a high degree of fluorescence resonance energy transfer (FRET), whereas displacement of the tracer with a kinase inhibitor results in loss of FRET (Table 7). Compounds **4d**, **8b**, **8e** and **8f** showed low nanomolar AAK1 affinity in this *in vitro* assay. Compound **7a** showed no AAK1 affinity, in agreement with the co-crystal X-ray structure of AAK1 with a closely related pyrrolo[2,3-*d*]pyridine (Verdonck et al., 2019), indicating that the pyrrole nitrogen is engaged in a critical hydrogen bond interaction with the kinase hinge region. Despite its lack of AAK1 affinity, compound **7a** displayed considerable anti-RSV-A activity, suggesting that the observed antiviral activity of this compound class is, at least in part, AAK1-independent. Nevertheless, it is possible that AAK1 inhibition plays a role in the observed antiviral effect.

**TABLE 7:** AAK1 inhibition.

Cmpd	AAK1 IC_50_ (µM)
**1**	0.0042
**4d**	0.0062
**7a**	>10
**8b**	0.0040
**8e**	0.0035
**8f**	0.0054

## 4 Evaluation of broad-spectrum antiviral activity

Because of the current interest in the development of novel drugs for the treatment of COVID-19, the complete library was also assessed for its potential activity against SARS-CoV-2-infected Vero E6 cells, using a CPE-based assay, with a high-content imaging (HCI) read-out (Persoons et al., 2021). GS-441524, the parent nucleoside of remdesivir, was included as positive control and reference compound. Whereas the large majority of compounds lacked anti-SARS-CoV-2 activity beyond toxicity, a small set of analogues was endowed with promising anti-SARS-CoV-2 activity (Table 8). All compounds that displayed anti-SARS-CoV-2 activity also displayed anti-RSV activity, whereas some compounds that displayed anti-RSV activity lacked anti-SARS-CoV-2 activity. Hence, the SAR of 3-alkynyl-5-aryl-7-aza-indoles as inhibitors of RSV and SARS-CoV-2 replication does not run in parallel.

**TABLE 8:** Anti-SARS-CoV-2 activity and cytotoxicity of selected 5-aryl-3-alkynyl-pyrrolo[2,3-*b*]pyridines.

Cmpd#	SARS-CoV-2 EC_50_ (µM)^a^	Vero E6 CC_50_ (µM)^b^
**GS-441524**	0.7 ± 0.07	72.9 ± 12.2
**8b**	0.58 ± 0.050	> 100
**8c**	6.03 ± 0.071	> 100
**8g**	0.76 ± 0.46	> 85
**8h**	2.13 ± 1.91	> 100
**8k**	10.20 ± 0.14	88.50 ± 1.41
**8L**	0.96 ± 0.32	6.46 ± 1.75
**9c**	1.82 ± 0.83	> 100

^a^ concentration that gives 50% rescue of the virus-reduced eGFP signals relative to untreated virus-infected control cells.

^b^ 50% cytotoxic concentration, as measured *via* the colorimetric formazan-based MTS cell viability assay.

To define the antiviral potential of this series of compounds beyond respiratory infections, we explored the broad-spectrum potency of the compounds against another non-related virus family, i.e., encephalitis-inducing alphaviruses. Thus, our 7-aza-indole library was also screened for its activity against the live-attenuated TC-83 vaccine strain of VEEV in U-87 MG cells (human brain astrocytes), using ML-336 as a positive control (Schroeder et al., 2014). The hit compound **1** (EC_50_ **=** 1.22 µM), as well as compounds **4e** (EC_50_ = 1.50 µM)**, 5f** (EC_50_ = 4.30 µM) and **7b** (EC_50_ = 3.92 µM) displayed potent activity against VEEV and no cytotoxicity for the U-87 MG cells, comparable to the antiviral activity we have recently reported with the pan-NAK inhibitor 5Z-7-oxozeanol (Table 9) (Pu et al., 2020). This is in contrast with the potent cytotoxicity observed for these analogues in the HEp-2 cells used for RSV-A screening. These findings reveal a strong dependency of cytotoxicity values on the cell lines and that the SAR for anti-RSV, anti-SARS-CoV-2 and anti-VEEV activity is different. Follow-up studies with the use of primary cell cultures (e.g., 3D lung epithelial Air Liquid Interface cell model) can explore the cytotoxicity and antiviral activity of the compounds in a more physiologically relevant *ex vivo* model. The majority of the active anti-VEEV compounds carry a carboxamide substituent on the 5-phenyl moiety. This substitution pattern also imparts good activity against RSV and SARS-CoV-2, and it can thus be considered as a privileged moiety for antiviral activity.

**TABLE 9:** Anti-VEEV activity and cytotoxicity of selected 5-aryl-3-alkynyl-pyrrolo[2,3-*b*]pyridines.

Cmpd	VEEV EC_50_ (µM)^a^	U-87 MG CC_50_ (µM)^b^
**1**	1.22 ± 0.22	>10
**4e**	1.50 ± 0.17	>10
**5f**	4.30 ± 0.47	>10
**7b**	3.92 ± 1.15	>10
**8a**	2.72 ± 0.93	>10
**8c**	3.49	>10
**8f**	2.75 ± 1.22	>10
**8g**	3.75 ± 1.08	>10
**8h**	2.48 ± 1.27	>10
**8i**	1.29 ± 1.41	>10
**8j**	4.57 ± 4.87	>10
**9b**	1.39 ± 0.20	8.99 ± 1.60
**9c**	1.80 ± 0.32	>10
**5Z-7-Oxozeanol**	2.66 ± 0.13	>10
**ML-336**	0.04 ± 0.01	>10

^a^ 50% effective concentration, or concentration required to inhibit virus-induced cytopathogenicity in U-87 MG cells by 50%.

^b^ 50% cytotoxic concentration, or concentration required to reduce viability of U-87 MG cells by 50%.

## 5 Conclusion

A previously identified AAK1 inhibitor (compound **1**) with demonstrated antiviral activity against the flavivirus DENV (Verdonck et al., 2019), the filovirus EBOV (Verdonck et al., 2019), and the coronavirus SARS-CoV-2 (Karim et al., 2022) showed also activity against RSV, albeit with a low SI. A systematic SAR exploration of compound **1** focusing on structural variations at positions 1, 3 and 5 of the 7-aza-indole scaffold yielded various analogues with enhanced anti-RSV activity and no cytotoxicity. Moreover, selected congeners from this pyrrolo[2,3-*b*]pyridine library showed promising activity against SARS-CoV-2 and VEEV. Compound **9c** emerged as having an interesting profile, with good activity against RSV-A (EC_50_ = 0.18 µM), SARS-CoV-2 (EC_50_ = 1.82 µM) and VEEV (EC_50_ = 1.8 µM), and no cytotoxicity with CC_50_ values exceeding 100 μM, 50 µM and 10 μM, for the Vero E6, HEp-2, and U-87 MG cells, respectively. The original hit compound **1** and several congeners were shown to be potent inhibitors of AAK1. The broad-spectrum antiviral activity of these compounds might be partially due to AAK1 inhibition, although other viral or cellular proteins might also be involved in the antiviral activity of these 7-aza-indoles. Overall, the 3-alkynyl-5-aryl-7-aza-indole scaffold is a promising chemotype for the discovery of antiviral agents acting against various emerging viruses.

## 6 Experimental section

### 6.1 Chemistry

#### 6.1.1 General

For all reactions, analytical grade solvents were used. Argon was used to carry out reactions under an inert atmosphere. Melting points were recorded with a Stuart SMP20 melting point apparatus. ^1^H and ^13^C NMR spectra were recorded on a Bruker Avance 300 MHz instrument (^1^H NMR, 300 MHz; ^13^C NMR, 75 MHz), 500 MHz instrument (^1^H NMR, 500 MHz; ^13^C NMR, 125 MHz) or a 600 MHz instrument (^1^H NMR, 600 MHz; ^13^C NMR, 150 MHz, ^19^F NMR, 471 MHz), using tetramethylsilane as internal standard for ^1^H NMR spectra and DMSO-d_6_ (39.5 ppm) or CDCl_3_ (77.2 ppm) for ^13^C NMR spectra. Abbreviations used are s = singlet, d = doublet, t = triplet, q = quartet, m = multiplet, b = broad. Coupling constants are expressed in Hz. High resolution mass spectra were acquired on a quadrupole orthogonal acceleration time-of-flight mass spectrometer (Synapt G2 HDMS, Waters, Milford, MA). Samples were infused at 3 ml/min and spectra were obtained in positive or negative ionization mode with a resolution of 15,000 (FWHM) using leucine enkephalin as lock mass. Precoated aluminum sheets (Fluka silica gel/TLC-cards, 254 nm) were used for TLC. Column chromatography was performed on silica gel 0.060–0.200 mm, 60 (Acros Organics).

### 6.2 General procedure for the Sonogashira coupling

A solution of 5-bromo-3-iodo-1*H*-pyrrolo[2,3-*b*]pyridine **2** (1 equiv) and triethylamine (3 equiv) in THF, was degassed with a flow of argon for 5–10 min. Then, Pd(PPh_3_)_2_Cl_2_ (0.02 equiv) and CuI (0.01 equiv) were added and the mixture was allowed to reach 30°C. Subsequently, a solution of the appropriate acetylene (1.2 equiv) in THF was added slowly over a period of 30 min. The reaction was degassed a second time, filled with argon and stirred at 30°C overnight. After disappearance of the starting material as monitored by TLC, the volatiles were evaporated in *vacuo* and the crude residue was purified by silica gel flash chromatography. Compounds **3a**-**j** were made according to this procedure, from which the exact procedure for the preparation of compound **3a** is shown below. Procedures for the synthesis of compounds **3b**-**j** are available in the supporting information.

**5-Bromo-3-(pyridin-3-ylethynyl)-1*H*-pyrrolo[2,3-*b*]pyridine (3a)**. This compound was obtained using 3-ethynylpyridine. The crude residue was purified by flash chromatography using a mixture of hexane and acetone (in a ratio of 4:1) as mobile phase, affording the title compound as a beige solid in 80% yield (73.6 mg, 0.25 mmol). ^1^H NMR (600 MHz, DMSO) *δ*: 7.46 (ddd, *J* = 7.9, 4.8, 0.8 Hz, 1H, arom H), 7.99–8.04 (m, 1H, arom H), 8.06 (s, 1H, arom H), 8.38–8.43 (s, 2H, arom H), 8.56 (dd, *J* = 4.9, 1.7 Hz, 1H, arom H), 8.81 (dd, *J* = 2.1, 0.8 Hz, 1H, arom H), 12.48 (s, 1H, NH) ppm. HR-MS m/z [M + H]^+^ calcd for C_14_H_8_BrN_3_ 297.9975, found 297.9975.

### 6.3 General procedure for *N*-alkylation of the pyrrolo[2,3-*b*]pyridine scaffold (6a-d)

To a solution of 5-bromo-3-(pyridin-3-ylethynyl)-1*H*-pyrrolo[2,3-*b*]pyridine **3a** (1 equiv) in dry THF, NaH 60% (2 equiv) was added and the mixture was stirred for 10–15 min at room temperature. The reaction was cooled at 0°C and the appropriate alkyl iodide, alkyl bromide or benzyl bromide (1.5 equiv) was added. The reaction mixture was stirred at room temperature overnight. After disappearance of the starting material as monitored by TLC, the volatiles were evaporated in *vacuo* and the crude residue was purified by silica gel flash chromatography. Compounds **6a**-**d** were made according to this procedure, from which the detailed procedure for compound **6a** is shown below. Procedures for the synthesis of compounds **6b**-**d** can be found in the supporting information.

**5-Bromo-1-methyl-3-(pyridin-3-ylethynyl)-1*H*-pyrrolo[2,3-*b*]pyridine (6a)**. This compound was obtained using methyl iodide. The crude residue was purified by flash chromatography using a mixture of hexane and acetone (in a ratio of 4:1) as mobile phase, affording the title compound as a beige solid in 91% yield (47.6 mg, 0.15 mmol). ^1^H NMR (600 MHz, CDCl_3_) *δ:* 3.90 (s, 3H, CH_3_), 7.29 (ddd, *J* = 7.9, 4.9, 0.8 Hz, 1H, arom H), 7.49 (s, 1H, arom H), 7.78–7.84 (m, 1H, arom H), 8.20 (d, *J* = 2.1 Hz, 1H, arom H), 8.41 (d, *J* = 2.1 Hz, 1H, arom H), 8.54 (dd, *J* = 4.9, 1.6 Hz, 1H, arom H), 8.76–8.79 (m, 1H, arom H) ppm. HR-MS m/z [M + H]^+^ calcd for C_15_H_10_BrN_3_ 312.0131, found 312.0135.

### 6.4 General procedure for Suzuki coupling at position five of the pyrrolo[2,3-*b*]pyridine scaffold

A solution of the corresponding 5-bromo-3-alkynyl-1*H*-pyrrolo[2,3-*b*]pyridine (1 equiv) in a mixture of dioxane/water (ratio 9:1) was degassed with argon and subsequently, the corresponding boronic acid (1.2 equiv), Pd(PPh_3_)_4_ (0.02 equiv) and K_2_CO_3_ (2 equiv) were added. The mixture was degassed a second time, filled with argon and stirred at reflux overnight. After completion of the reaction as monitored by TLC, the volatiles were evaporated to dryness and the resulting residue was purified by silica gel and precipitate with diethyl ether yielding the title compounds. Compounds **4a**-**l**, **5a**-**e**, **7a**-**d**, **8a**-**m** and **9a-g** were made according to this procedure. The exact protocol for the synthesis of compound **4a** is shown, whereas the synthesis of the other derivatives is explained in the supporting information.

**5-(4-Methoxyphenyl)-3-(pyridin-3-ylethynyl)-1*H*-pyrrolo[2,3-*b*]pyridine (4a)**. This compound was obtained using the precursor **3a** and 4-methoxyphenylboronic acid. The crude residue was purified by flash chromatography using a mixture of hexane and acetone (in a ratio of 4:1) as mobile phase, affording the title compound as a white solid in 78% yield (42.5 mg, 0.13 mmol). ^1^H NMR (500 MHz, DMSO) *δ*: 3.81 (s, 3H, OCH_3_), 7.05 (d, *J* = 8.7 Hz, 2H, arom H), 7.45 (dd, *J* = 7.8, 4.9 Hz, 1H, arom H), 7.72 (d, *J* = 8.7 Hz, 2H, arom H), 7.97–8.03 (m, 2H, arom H), 8.26 (d, *J* = 2.1 Hz, 1H, arom H), 8.54 (dd, *J* = 4.8, 1.4 Hz, 1H, arom H), 8.58 (d, *J* = 2.0 Hz, 1H, arom H), 8.80 (d, *J* = 1.7 Hz, 1H, arom H), 12.28 (s, 1H, NH) ppm. ^13^C NMR (150 MHz, DMSO) *δ*: 55.3 (OCH_3_), 86.8 (C), 87.7 (C), 94.7 (C), 114.6 (CH), 120.3 (C), 120.5 (C), 123.6 (CH), 124.8 (CH), 128.3 (CH), 129.3 (C), 130.8 (C), 131.7 (CH), 138.1 (CH), 142.9 (CH), 147.2 (C), 148.3 (CH), 151.4 (CH), 158.9 (C) ppm. HR-MS m/z [M + H]^+^ calcd for C_21_H_15_N_3_O 326.1288, found 326.1289.

### 6.5 Cells

HEp-2, A549, and U-87 MG cells were obtained from the American Type Culture Collection (ATCC). HEp-2 cells were grown in Dulbecco’s modified eagle medium (DMEM; Gibco cat no 41965-039) supplemented with heat-inactivated 8% (v/v) fetal bovine serum (FBS, Hyclone). A549 cells were cultured in DMEM (Gibco) supplemented with 10% (v/v) FBS (Hyclone), 0.075% (m/v) NaHCO3 (Gibco), and 1 mM sodium pyruvate (Gibco). U-87 MG cells were grown in DMEM supplemented with 10% FBS and 1X penicillin-streptomycin solution. Cells were maintained in a humidified incubator with 5% CO_2_ at 37°C. Cells were cultured at 37°C in a humified environment with 5% CO_2_, and passaged every 3–4 days.

VeroE6-eGFP cells (provided by Dr. K. Andries, J&JPRD, Beerse, Belgium) were maintained in DMEM (Gibco cat no 41965-039) supplemented with heat-inactivated 10% FBS and 500 μg/ml Geneticin (Gibco cat no 10131-0275) and kept under 5% CO_2_ at 37°C.

### 6.6 Viruses

RSV-A strain Long was purchased from ATCC and propagated in HEp-2 cells in DMEM (Gibco) supplemented with 10% inactivated FBS (Biowest). The infectious content of the virus stock was determined by end-point titration on HEp-2 cells.

The SARS-CoV-2 isolate used in this study was the BetaCov/Belgium/GHB-03021/2020 (EPI ISL407976|2020-02-03), which was isolated from a Belgian patient returning from Wuhan in February 2020. The isolate was passaged 7 times on VeroE6 cells which introduced two series of amino acid deletions in the spike protein (Boudewijns et al., 2020). The infectious content of the virus stock was determined by end-point titration on Vero E6 cells.

All SARS-CoV2-related experimental work was performed in the certified, high-containment biosafety level-3 facilities of the Rega Institute at the KU Leuven.

The plasmid encoding infectious VEEV (TC-83) with a nanoluciferase reporter which was used for virus production (VEEV-TC-83-nLuc) was a gift from Dr. William B. Klimstra (Department of Immunology, University of Pittsburgh) (Sun et al., 2014). The VEEV-TC-83-nLuc virus was harvested from the supernatant 24 h post-electroporation, clarified from cell debris and the titer determined by standard plaque assay on Vero cells.

### 6.7 RSV screening

The RSV antiviral assay is based on a cytopathic effect (CPE)-reduction assay. Briefly, HEp-2 cells were seeded in 96-well microtiter plates in cell culture medium at a density of 15,000 cells/well and allowed to adhere overnight. The following day, 5-fold serial dilutions of test compounds were prepared in cell infectious medium (cell culture medium with 2% FBS) and added to the semi-confluent cell monolayers. Next, cells were either exposed to RSV-A virus (MOI = 0.01) or mock-infected (cytotoxicity screening), and incubated at 37°C, 5% CO_2_ for 4 days. The virus-induced CPE was microscopically evaluated and a MTS-PES assay was performed. The reference compounds ribavirin, DS 10,000, and TMC353121 were included as controls. All conditions were performed in duplicate.

A549 cells were seeded in 96-well microtiter plates in cell culture medium at a density of 20,000 cells/well and allowed to adhere overnight. The following day, 5-fold serial dilutions of test compounds were prepared in cell infectious medium (cell culture medium with 2% FBS) and added to the semi-confluent cell monolayers. Next, cells were either exposed to RSV-A virus (MOI = 0.1) or mock-infected (cytotoxicity screening) for 2 h, washed with PBS to remove unbound virus, followed by incubation with freshly prepared 5-fold serial dilutions of compounds or cell infectious medium at 37°C under 5% CO_2_ for 5 days. Supernatant was harvested and stored at −20°C for subsequent analysis of viral copy numbers with reverse transcription quantitative PCR (RT-qPCR).

In parallel, toxicity of compounds in the absence of virus was evaluated in a standard MTS-assay.

### 6.8 Viability assays

The viability of virus- and mock-infected Vero E6 and A549 cells was spectrophotometrically evaluated based on the *in situ* reduction of the MTS tetrazolium compound [3-(4,5-dimethylthiazol-2-yl)-5-(3-carboxy-methoxyphenyl)-2-(4-sulfophenyl)-2H tetrazolium] by metabolically active cells. For each well of the 96-well assay plate, 50 µl MTS/PES solution (1.33 mg/ml CellTiter 96^®^ AQ_ueous_ MTS Reagent Powder [Promega], 0.21 mM phenazine ethosulfaat [PES, MP Biomedicals]) in PBS) was added to 100 µl supernatant. After incubating for 2 h at 37°C and 5% CO_2_, 50 µl of 0.1% Triton X-100 (Thermo Scientific) was added, followed by absorbance measurement at 490 nm using the SpectraMax 384 Plus Microplate Reader (Molecular Devices). To calculate the compound concentration required to inhibit virus-induced cell death by 50%, the obtained optical density (OD) values were compared with a cell control condition (cells in absence of virus and compound) and a virus control condition (cells in presence of virus but without compound)*.*

Viability of U-87 MG VEEV-infected cells was measured using alamarBlue^®^ reagent (ThermoFisher Scientific, Waltham, MA, United States) according to the manufacturer’s protocol. Fluorescence was detected at 560 nm on InfiniteM1000 plate reader (Tecan, Männedorf, Switzerland). The raw fluorescence values were normalized to DMSO-treated cells (set as 100%). GraphPad Prism nonlinear regression (curve fit) was used to generate the graphs and CC_50_ values.

### 6.9 SARS-CoV-2 screening

The SARS-CoV-2 antiviral assay is derived from the previously established SARS-CoV assay (Ivens et al., 2005). In this assay, fluorescence of VeroE6-eGFP cells (provided by Dr. K. Andries J&JPRD; Beerse, Belgium) declines after infection with SARS-CoV-2 due to the cytopathogenic effect of the virus. In the presence of an antiviral compound, the cytopathogenicity is inhibited and the fluorescent signal maintained.

Stock solutions of the various compounds in DMSO (10 mM) were prepared. On day -1, VeroE6-GFP cells were seeded at a density of 25,000 cells/well in 96-well plates (Greiner Bio One, catalog no. 655090) and pretreated with threefold serial dilutions of the compounds. Serial dilutions were performed in assay medium (DMEM supplemented with 2% v/v FCS). The plates were incubated (37 °C, 5% CO_2_ and 95% relative humidity) overnight. On the next day (day 0), cells were infected with the SARS-CoV-2 inoculum at a multiplicity of infection (MOI) of 0.001 tissue culture infectious dose (TCID50) per cell. The plates were incubated in a humidified incubator at 37°C and 5% CO_2_. At 4 days p.i., the wells were examined for eGFP expression using an argon laser-scanning microscope. The microscope settings were excitation at 488 nm and emission at 510 nm and the fluorescence images of the wells were converted into signal values. The results were expressed as EC_50_ values defined as the concentration of compound achieving 50% inhibition of the virus-reduced eGFP signals as compared to the untreated virus-infected control cells. Toxicity of compounds in the absence of virus was evaluated in a standard MTS-assay as described previously (Jochmans et al., 2012).

### 6.10 VEEV (TC-83) screening

U-87 MG cells were treated with the inhibitors or DMSO. One hour later, the cells were infected with VEEV-TC-83-nLuc virus in five replicates at MOI of 0.01, and overall infection was measured at 18 h post-infection *via* a nanoluciferase assay. The inhibitors were present for the duration of the experiment. The relative light units (RLUs) were normalized to DMSO-treated cells (set as 100%). GraphPad Prism nonlinear regression (curve fit) was used to generate the graphs and EC_50_ values. The complete set of antiviral data against VEEV is available in the Supplementary Material.

### 6.11 RNA extraction and qPCR

Lysis of viral particles and RNA extraction were preformed using the QIAamp Viral RNA Mini Kit (QIAGEN) according to the manufacturer’s instructions. An RT-qPCR assay to detect the N gene of RSV-A was performed using the QuantStudio™ 5 Real-Time PCR System (Applied Biosystems). For the detection of the RSV-A N gene, each reaction (20 µl) contained 5 µl of sample, 1X TaqMan Fast Virus 1-step MasterMix 4X (Applied Biosystems), 0.2 µM of both forward (5′-TGC​ACA​CTA​GCA​TGT​CCT​AAC-3′) and reverse (5′-GGC​AGT​AGA​GTT​GAA​GGG​ATT​T-3′) primers, and 0.1 µM probe (5′-TGC​CCT​GCA​CCA​TAG​GCA​TTC​ATA-3′). All primers and probes were obtained from Integrated DNA Technologies (IDT). The following PCR run conditions were used: 1 cycle of 15 min at 50°C and 2 min at 95°C followed by 40 cycles of 15 s at 95°C and 45 s at 60°C. Data analysis was performed using the QuantStudio™ Design & Analysis Software (version v1.5.2, Applied Biosystems). A standard curve was produced using 10-fold serial dilutions of plasmid DNA with known concentrations. All samples were run in duplicate.

## Data Availability

The original contributions presented in the study are included in the article/Supplementary Material, further inquiries can be directed to the corresponding author.
