# Supplementary material for: Synthesis and evaluation of 3-alkynyl-5-aryl-7-aza-indoles as broad-spectrum antiviral agents
Source: Front Chem. 2022 Oct 26;10:1058229. doi: 10.3389/fchem.2022.1058229 (PMC9643853; doi:10.3389/fchem.2022.1058229)
Supplement: Supplementary file 1 [file DataSheet1.PDF]

Supporting information

Synthesis and evaluation of 3-alkynyl-5-aryl-7-aza-indoles as broad-spectrum antiviral agents

Belén Martinez-Gualda,[1,2] Mirthe Graus,[2] Anita Camps,[2] Emiel Vanhulle,[2] Sirle Saul,[3] Siavash Azari,[3] Do Hoang Nhu Tran,[3] Laura Vangeel,[2] Winston Chiu,[2] Johan Neyts,[2] Dominique Schols,[2] Shirit Einav,[3,4] Kurt Vermeire,[2†] Steven De Jonghe[2†]

[1]KU Leuven, Department of Pharmaceutical and Pharmacological Sciences, Rega Institute for Medical Research, Laboratory of Medicinal Chemistry, Herestraat 49, box 1041, 3000 Leuven, Belgium

[2]KU Leuven, Department of Microbiology, Immunology and Transplantation, Rega Institute for Medical Research, Laboratory of Virology and Chemotherapy, Herestraat 49, box 1043, 3000 Leuven, Belgium

[3]Department of Medicine, Division of Infectious Diseases and Geographic Medicine, and Department of Microbiology and Immunology, Stanford University, CA, USA

[4]Chan Zuckerberg Biohub, San Francisco, CA, USA

†These authors contributed equally to this work and share last authorship. Corresponding authors : kurt.vermeire@kuleuven.be and steven.dejonghe@kuleuven.be

Contents

Experimental procedures for synthesis of compounds **3b-j**, **6b-d**, **4b-k**, **5b-f**, **7a-d**, **8a-m** and **9a-g**

Anti-VEEV activity of the 7-aza-indole compound library: Tables S1-S5

Sonogashira couplings

5-Bromo-3-(pyridin-2-ylethynyl)-1*H*-pyrrolo[2,3-*b*]pyridine (3b). This compound was obtained using 2-ethynylpyridine and the reaction mixture was stirred at 70 ºC overnight. The crude residue was purified by flash chromatography using a mixture of hexane and acetone (in a ratio of 4:1) as mobile phase, affording the title compound as a beige solid in 24% yield (30.5 mg, 0.10 mmol). ^{1}H NMR (500 MHz, DMSO) *δ*: 7.37 (dd, *J* = 6.9, 5.0 Hz, 1H, arom H), 7.71 (d, *J* = 7.8 Hz, 1H, arom H), 7.84 (td, *J* = 7.7, 1.7 Hz, 1H, arom H), 8.11 (s, 1H, arom H), 8.32 (d, *J* = 2.2 Hz, 1H, arom H), 8.41 (d, *J* = 2.1 Hz, 1H, arom H), 8.59 (d, *J* = 4.7 Hz, 1H, arom H), 12.54 (s, 1H, NH) ppm. HR-MS m/z $[M+H]^+$ calcd for $C_{14}H_8BrN_3$ 297.9975, found 297.9979.

5-Bromo-3-(phenylethynyl)-1*H*-pyrrolo[2,3-*b*]pyridine (3c). This compound was obtained using phenylacetylene. The crude residue was purified by flash chromatography using a mixture of hexane and acetone (in a ratio of 4:1) as mobile phase, affording the title compound as a beige solid in 81% yield (73.6 mg, 0.25 mmol). ^{1}H NMR (300 MHz, DMSO) *δ:* 7.37–7.47 (m, 3H, arom H), 7.56–7.64 (m, 2H, arom H), 8.01 (s, 1H, arom H), 8.33 (d, *J* = 2.1 Hz, 1H, arom H), 8.39 (d, *J* = 2.2 Hz, 1H, arom H), 12.43 (s, 1H, NH) ppm. HR-MS m/z $[M+H]^+$ calcd for $C_{15}H_9BrN_2$ 297.0022, found 297.0029.

5-Bromo-3-(4-(fluorophenyl)ethynyl)-1*H*-pyrrolo[2,3-*b*]pyridine (3d). This compound was obtained using 4-fluorophenylacetylene. The crude residue was purified by flash chromatography using a mixture of hexane and ethyl acetate (in a ratio of 7:3) as mobile phase, affording the title compound as a beige solid in 85% yield (82.7 mg, 0.26 mmol). ^{1}H NMR (300 MHz, DMSO) *δ:* 7.22–7.33 (m, 2H, arom H), 7.62–7.71 (m, 2H, arom H), 8.00 (s, 1H, arom H), 8.35 (d, *J* = 2.1 Hz, 1H, arom H), 8.39 (d, *J* = 2.1 Hz, 1H, arom H), 12.43 (s, 1H, NH). ppm. HR-MS m/z $[M+H]^+$ calcd for $C_{15}H_8BrFN_2$ 314.9928, found 314.9931.

5-Bromo-3-(3-(chlorophenyl)ethynyl)-1*H*-pyrrolo[2,3-*b*]pyridine (3e). This compound was obtained using 3-chlororophenylacetylene. The crude residue was purified by flash chromatography using a mixture of hexane and ethyl acetate (in a ratio of 4:1) as mobile phase, affording the title compound as a beige solid in 55% yield (56.5 mg, 0.17 mmol). ^{1}H NMR (300 MHz, DMSO) *δ:* 7.43–7.47 (m, 2H, arom H), 7.58–7.52 (m, 2H, arom H), 7.73 (bs, 1H, arom H), 8.03 (s, 1H, arom H), 8.39 (d, *J* = 2.1 Hz, 1H, arom H), 8.43 (d, *J* = 2.1 Hz, 1H, arom H),

12.48 (bs, 1H, NH) ppm. HR-MS m/z $[M+H]^+$ calcd for $C_{15}H_8BrClN_2$ 330.9633, found 330.9626.

5-Bromo-3-(3-(methoxyphenyl)ethynyl)-1*H*-pyrrolo[2,3-*b*]pyridine (3f). This compound was obtained using 3-methoxyphenylacetylene. The crude residue was purified by flash chromatography using a mixture of hexane and ethyl acetate (in a ratio of 4:1) as mobile phase, affording the title compound as a white solid in 76% yield (69.9 mg, 0.24 mmol). ^{1}H NMR (300 MHz, $CDCl_3$) δ: 3.85 (s, 3H, OCH_3), 6.91 (ddd, *J* = 8.2, 2.5, 0.8 Hz, 1H, arom H), 7.09 (dd, *J* = 2.3, 1.3 Hz, 1H, arom H), 7.16 (d, *J* = 7.6 Hz, 1H, arom H), 7.25–7.32 (m, 1H, arom H), 7.63 (d, *J* = 2.6 Hz, 1H, arom H), 8.28 (d, *J* = 2.0 Hz, 1H, arom H), 8.42 (d, *J* = 2.1 Hz, 1H, arom H), 10.33 (s, 1H, NH) ppm. HR-MS m/z $[M+H]^+$ calcd for $C_{16}H_{11}BrN_2O$ 327.0128, found 327.0127.

5-Bromo-3-(thiophen-3-ylethynyl)-1*H*-pyrrolo[2,3-*b*]pyridine (3g). This compound was obtained using 3-ethynylthiophene. The crude residue was purified by flash chromatography using a mixture of hexane and ethyl acetate (in a ratio of 4:1) as mobile phase, affording the title compound as a light yellow solid in 74% yield (69.4 mg, 0.23 mmol). ^{1}H NMR (300 MHz, DMSO) δ: 7.31 (d, *J* = 5.0 Hz, 1H, arom H), 7.62–7.66 (m, 1H, arom H), 7.85–7.89 (m, 1H, arom H), 7.98 (s, 1H, arom H), 8.27 (d, *J* = 1.5 Hz, 1H, arom H), 8.38 (d, *J* = 1.5 Hz, 1H, arom H), 12.40 (s, 1H, NH) ppm. HR-MS m/z $[M+H]^+$ calcd for $C_{13}H_7BrN_2S$ 302.9587, found 302.9587.

5-Bromo-3-((5-methylpyridin-3-yl)ethynyl)-1*H*-pyrrolo[2,3-*b*]pyridine (3h). This compound was obtained using 3-ethynyl-5-methoxypyridine. The crude residue was purified by flash chromatography using a mixture of hexane and acetone (in a ratio of 4:1) as mobile phase, affording the title compound as a beige solid in 63% yield (60.9 mg, 0.20 mmol). ^{1}H NMR (300 MHz, DMSO) δ: 2.33 (s, 3H, CH_3), 7.85 (s, 1H, arom H), 8.05 (d, *J* = 2.4 Hz, 1H, arom H), 8.37–8.42 (m, 3H, arom H), 8.60 (s, 1H, arom H), 12.48 (s, 1H, NH) ppm. HR-MS m/z $[M+H]^+$ calcd for $C_{15}H_{10}BrN_3$ 312.0131, found 321.0126.

5-Bromo-3-((6-fluoropyridin-3-yl)ethynyl)-1H-pyrrolo[2,3-b]pyridine (3i). This compound was obtained using 5-ethynyl-2-fluoropyridine. The crude residue was purified by flash chromatography using a mixture of hexane and acetone (in a ratio of 4:1) as mobile phase, affording the title compound as a beige solid in 72% yield (70.4 mg, 0.22 mmol). ^{1}H NMR (300 MHz, DMSO) δ: 7.29 (dd, *J* = 8.5, 2.7 Hz, 1H, arom H), 8.05 (d, *J* = 2.7 Hz, 1H, arom H), 8.22

(td, *J* = 8.2, 2.3 Hz, 1H, arom H), 8.40 (s, 2H, arom H), 8.51 (s, 1H, arom H), 12.48 (s, 1H, NH) ppm. HR-MS m/z $[M+H]^+$ calcd for $C_{14}H_7BrFN_3$ 315.9881, found 315.9882.

5-Bromo-3-((5-methoxypyridin-3-yl)ethynyl)-1*H*-pyrrolo[2,3-*b*]pyridine (3j). This compound was obtained using 3-ethynyl-5-methoxypyridine. The crude residue was purified by flash chromatography using a mixture of hexane and acetone (in a ratio of 4:1) as mobile phase, affording the title compound as a beige solid in 68% yield (69.1 mg, 0.21 mmol). ^1H NMR (300 MHz, DMSO) δ: 3.88 (s, 3H, OCH_3), 7.62 (bs, 1H, arom H), 8.05 (d, *J* = 2.6 Hz, 1H, arom H), 8.28 (bs, 1H, arom H), 8.37–8.42 (m, 3H, arom H), 12.49 (s, 1H, NH) ppm. HR-MS m/z $[M+H]^+$ calcd for $C_{15}H_{10}BrN_3O$ 328.0080, found 328.0080.

Synthesis of compounds 6b-d

5-Bromo-1-ethyl-3-(pyridin-3-ylethynyl)-1*H*-pyrrolo[2,3-*b*]pyridine (6b). This compound was obtained using ethyl bromide. The crude residue was purified by flash chromatography using a mixture of hexane and acetone (in a ratio of 4:1) as mobile phase, affording the title compound as a beige solid in 84% yield (45.9 mg, 0.14 mmol). ^1H NMR (300 MHz, $CDCl_3$) δ: 1.49 (t, *J* = 7.3 Hz, 3H, CH_3), 4.33 (q, *J* = 7.3 Hz, 2H, CH_2), 7.28 (dd, *J* = 7.5, 4.6 Hz, 1H, arom H), 7.54 (s, 1H, arom H), 7.80 (dt, *J* = 7.9, 1.8 Hz, 1H, arom H), 8.18 (d, *J* = 2.1 Hz, 1H, arom H), 8.39 (d, *J* = 2.1 Hz, 1H, arom H), 8.53 (dd, *J* = 4.8, 1.5 Hz, 1H, arom H), 8.77 (d, *J* = 1.4 Hz, 1H, arom H) ppm. HR-MS m/z $[M+H]^+$ calcd for $C_{16}H_{12}BrN_3$ 326.0288, found 326.0285.

5-Bromo-1-isobutyl-3-(pyridin-3-ylethynyl)-1*H*-pyrrolo[2,3-*b*]pyridine (6c). To a solution of 5-bromo-3-(pyridin-3-ylethynyl)-1*H*-pyrrolo[2,3-*b*]pyridine **2a** (1 equiv) in DMF, ^{t}BuOK (2 equiv) was added and the mixture was stirred for 10-15 min at room temperature. After this time, isobutyl iodide (1.5 equiv) was added and the reaction mixture was stirred at 110 ºC overnight. After disappearance of the starting material as monitored by TLC, the volatiles were evaporated in *vacuo* and the crude residue was purified by silica gel flash chromatography using a mixture of hexane and ethyl acetate (in a ratio of 4:1) as mobile phase, affording the title compound as a beige solid in 79% yield (46.9 mg, 0.13 mmol). ^1H NMR (300 MHz, $CDCl_3$) δ: 0.93 (d, *J* = 6.7 Hz, 6H, 2 x CH_3), 2.18-2.33 (m, 1H, CH), 4.08 (d, *J* = 7.4 Hz, 2H, CH_2), 7.29 (dd, *J* = 7.9, 5.0 Hz, 1H, arom H), 7.50 (s, 1H, arom H), 7.82 (dt, *J* = 7.9, 1.8 Hz, 1H, arom H), 8.20 (d, *J* = 2.1 Hz, 1H, arom H), 8.39 (d, *J* = 2.1 Hz, 1H, arom H), 8.54 (dd, *J* = 4.8, 1.4 Hz, 1H, arom H), 8.78 (d, *J* = 1.5 Hz, 1H, arom H) ppm. HR-MS m/z $[M+H]^+$ calcd for $C_{18}H_{16}BrN_3$ 354.0601, found 354.0600.

1-Benzyl-5-bromo-3-(pyridin-3-ylethynyl)-1*H*-pyrrolo[2,3-*b*]pyridine (6d). This compound was obtained using benzyl bromide. The crude residue was purified by flash chromatography using a mixture of hexane and acetone (in a ratio of 4:1) as mobile phase and a second time using dichloromethane and ethyl acetate (9:1), affording the title compound as a light yellow solid in 83% yield (54.0 mg, 0.14 mmol). ^{1}H NMR (300 MHz, $CDCl_3$) *δ:* 5.47 (s, 2H, CH_2), 7.24–7.36 (m, 6H, arom H), 7.48 (s, 1H, arom H), 7.79 (dt, *J* = 7.9, 1.8 Hz, 1H, arom H), 8.22 (d, *J* = 2.1 Hz, 1H, arom H), 8.43 (d, *J* = 2.1 Hz, 1H, arom H), 8.52–8.55 (m, 1H, arom H), 8.76 (bs, 1H, arom H) ppm. HR-MS m/z $[M+H]^+$ calcd for $C_{21}H_{14}BrN_3$ 388.0444, found 388.0438.

Suzuki couplings

5-(3-Methoxyphenyl)-3-(pyridin-3-ylethynyl)-1*H*-pyrrolo[2,3-*b*]pyridine (4b). This compound was obtained using the precursor **3a** and 3-methoxyphenylboronic acid. The crude residue was purified by flash chromatography using a mixture of hexane and acetone (in a ratio of 4:1) as mobile phase, affording the title compound as a white solid in 76% yield (41.4 mg, 0.13 mmol). ^{1}H NMR (600 MHz, DMSO) *δ*: 3.86 (s, 3H, OCH_3), 6.97 (ddd, *J* = 8.2, 2.6, 0.8 Hz, 1H, arom H), 7.31– 7.33 (m, 1H, arom H), 7.34–7.37 (m, 1H, arom H), 7.42 (t, *J* = 7.9 Hz, 1H, arom H), 7.46 (ddd, *J* = 7.9, 4.8, 0.9 Hz, 1H, arom H), 8.01–8.03 (m, 1H, arom H), 8.04 (s, 1H, arom H), 8.35 (d, *J* = 2.2 Hz, 1H, arom H), 8.56 (dd, *J* = 4.8, 1.6 Hz, 1H, arom H), 8.64 (d, *J* = 2.2 Hz, 1H, arom H), 8.82 (dd, *J* = 2.1, 0.8 Hz, 1H, arom H), 12.34 (s, 1H, NH) ppm. ^{13}C NMR (150 MHz, DMSO) *δ:* 55.3 (OCH_3), 86.7 (C), 87.7 (C), 94.9 (C), 112.7 (CH), 113.0 (CH), 119.6 (CH), 120.3 (C), 120.5 (C), 123.6 (CH), 125.5 (CH), 129.5 (C), 130.2 (CH), 131.9 (CH), 138.2 (CH), 140.0 (C), 143.3 (CH), 147.5 (C), 148.3 (CH), 151.4 (CH), 159.9 (C) ppm. HR-MS m/z $[M+H]^+$ calcd for $C_{21}H_{15}N_3O$ 326.1288, found 326.1292.

5-(2-Methoxyphenyl)-3-(pyridin-3-ylethynyl)-1*H*-pyrrolo[2,3-*b*]pyridine (4c). This compound was obtained using the precursor **2a** and 2-methoxyphenylboronic acid. The crude residue was purified by flash chromatography using a mixture of hexane and acetone (in a ratio of 4:1) as mobile phase, affording the title compound as a white solid in 75% yield (40.8 mg, 0.13 mmol). ^{1}H NMR (600 MHz, DMSO) *δ:* 3.79 (s, 3H, OCH_3), 7.07 (td, *J* = 7.4, 1.0 Hz, 1H, arom H), 7.16 (dd, *J* = 8.2, 0.7 Hz, 1H, arom H), 7.37–7.44 (m, 3H, arom H), 7.96–7.99 (m, 1H, arom H), 8.00 (s, 1H, arom H), 8.13 (d, *J* = 2.1 Hz, 1H, arom H), 8.40 (d, *J* = 2.1 Hz, 1H, arom H), 8.53 (dd, *J* = 4.8, 1.6 Hz, 1H, arom H), 8.76 (dd, *J* = 2.2, 0.9 Hz, 1H, arom H), 12.28 (bs, 1H, NH) ppm. ^{13}C NMR (150 MHz, DMSO) *δ:* 55.7 (OCH_3), 86.7 (C), 87.6 (C), 94.6 (C),

111.8 (CH), 119.8 (C), 120.5 (C), 121.0 (CH), 123.6 (CH), 127.2 (C), 127.7 (CH), 127.7 (C), 129.1 (CH), 131.1 (CH), 131.4 (CH), 138.1 (CH), 145.1 (CH), 146.9 (C), 148.3 (CH), 151.3 (CH), 156.4 (C) ppm. HR-MS m/z $[M+H]^+$ calcd for $C_{21}H_{15}N_3O$ 326.1288, found 326.1290.

5-(3,5-Dimethoxyphenyl)-3-(pyridin-3-ylethynyl)-1*H*-pyrrolo[2,3-*b*]pyridine (4d). This compound was obtained using the precursor **3a** and 3,5-dimethoxyphenylboronic acid. The crude residue was purified by flash chromatography using a mixture of hexane and acetone (in a ratio of 4:1) as mobile phase, affording the title compound as a white solid in 79% yield (47.1 mg, 0.13 mmol). ^{1}H NMR (600 MHz, DMSO) δ: 3.84 (s, 6H, 2 x OCH_3), 6.53 (t, J = 2.2 Hz, 1H, arom H), 6.90 (d, J = 2.3 Hz, 2H, arom H), 7.46 (ddd, J = 7.9, 4.8, 0.9 Hz, 1H, arom H), 7.97–8.06 (m, 2H, arom H), 8.34 (d, J = 2.2 Hz, 1H, arom H), 8.55 (dd, J = 4.8, 1.6 Hz, 1H, arom H), 8.64 (d, J = 2.2 Hz, 1H, arom H), 8.81 (dd, J = 2.2, 0.9 Hz, 1H, arom H) ppm. ^{13}C NMR (150 MHz, DMSO) δ: 55.4 (OCH_3), 86.7 (C), 87.7 (C), 94.9 (C), 99.3 (CH), 105.4 (CH), 120.2 (C), 120.5 (C), 123.6 (CH), 125.6 (CH), 129.6 (C), 132.0 (CH), 138.2 (CH), 140.7 (C), 143.4 (CH), 147.6 (C), 148.3 (CH), 151.4 (CH), 161.0 (C) ppm. HR-MS m/z $[M+H]^+$ calcd for $C_{22}H_{17}N_3O_2$ 356.1393, found 356.1394.

5-(3,4,5-Trimethoxyphenyl)-3-(pyridin-3-ylethynyl)-1*H*-pyrrolo[2,3-*b*]pyridine (4e). This compound was obtained using the precursor **2a** and 3,4,5-trimethoxyphenylboronic acid. The crude residue was purified by flash chromatography using a mixture of hexane and acetone (in a ratio of 7:3) as mobile phase, affording the title compound as a white solid in 75% yield (48.4 mg, 0.13 mmol). ^{1}H NMR (600 MHz, DMSO) δ: 3.71 (s, 3H, OCH_3), 3.91 (s, 6H, 2 x OCH_3), 7.02 (s, 2H, arom H), 7.46 (ddd, J = 7.9, 4.9, 0.9 Hz, 1H, arom H), 8.00–8.03 (m, 1H, arom H), 8.03 (s, 1H, arom H), 8.34 (d, J = 2.2 Hz, 1H, arom H), 8.55 (dd, J = 4.8, 1.6 Hz, 1H, arom H), 8.66 (d, J = 2.2 Hz, 1H, arom H), 8.81 (dd, J = 2.2, 0.8 Hz, 1H, arom H), 12.33 (s, 1H, NH) ppm. ^{13}C NMR (150 MHz, DMSO) δ: 56.2 (OCH_3), 60.2 (OCH_3), 86.8 (C), 87.7 (C), 94.8 (C), 104.9 (CH), 120.1 (C), 120.5 (C), 123.6 (CH), 125.5 (CH), 129.9 (C), 131.9 (CH), 134.3 (C), 137.1 (C), 138.2 (CH), 143.5 (CH), 147.4 (C), 148.3 (CH), 151.4 (CH), 153.4 (C) ppm. HR-MS m/z $[M+H]^+$ calcd for $C_{23}H_{19}N_3O_3$ 386.1499, found 386.1499.

5-(4-(2-Methoxyethoxy)phenyl)-3-(pyridin-3-ylethynyl)-1*H*-pyrrolo[2,3-*b*]pyridine (4f). This compound was obtained using the precursor **2a** and 4-(2-methoxyethoxy)phenylboronic acid. The crude residue was purified by flash chromatography using a mixture of hexane and acetone (in a ratio of 7:3) as mobile phase, affording the title compound as a white solid in 76% yield (47.1 mg, 0.13 mmol). ^{1}H NMR (600 MHz, DMSO) δ: 3.32 (s, 3H, OCH_3), 3.66–3.71 (m,

2H, OCH_2), 4.13–4.16 (m, 2H, OCH_2), 7.06 (d, *J* = 8.7 Hz, 2H, arom H), 7.45 (dd, *J* = 7.8, 4.8 Hz, 1H, arom H), 7.71 (d, *J* = 8.7 Hz, 2H, arom H), 7.98–8.03 (m, 2H, arom H), 8.27 (d, *J* = 2.1 Hz, 1H, arom H), 8.55 (dd, *J* = 4.8, 1.5 Hz, 1H, arom H), 8.59 (d, *J* = 2.1 Hz, 1H, arom H), 8.81 (d, *J* = 1.4 Hz, 1H, arom H), 12.27 (s, 1H, NH) ppm. ^{13}C NMR (150 MHz, DMSO) *δ:* 58.3 (CH_3), 67.1 (OCH_2), 70.5 (OCH_2), 86.7 (C), 87.7 (C), 94.7 (C), 115.1 (CH), 120.3 (C), 120.5 (C), 123.6 (CH), 124.8 (CH), 128.3 (CH), 129.3 (C), 130.9 (C), 131.7 (CH), 138.1 (CH), 142.9 (CH), 147.2 (C), 148.3 (CH), 151.4 (CH), 158.1 (C) ppm. HR-MS m/z $[M+H]^+$ calcd for $C_{23}H_{19}N_3O_2$ 370.1515, found 370.1538.

5-(2-Methoxypyridin-4-yl)-3-(pyridin-3-ylethynyl)-1*H*-pyrrolo[2,3-*b*]pyridine (4g). This compound was obtained using the precursor **3a** and 2-methoxypyridine-4-boronic acid. The crude residue was purified by flash chromatography using a mixture of hexane and acetone (in a ratio of 7:3) as mobile phase, affording the title compound as a white solid in 75% yield (41.0 mg, 0.13 mmol). ^1H NMR (600 MHz, DMSO) *δ:* 3.91 (s, 3H, OCH_3), 7.29 (s, 1H, arom H), 7.46 (dd, *J* = 7.7, 4.9 Hz, 1H, arom H), 7.48 (dd, *J* = 5.3, 1.1 Hz, 1H, arom H), 8.00–8.05 (m, 1H, arom H), 8.06 (s, 1H, arom H), 8.25 (d, *J* = 5.3 Hz, 1H, arom H), 8.49 (d, *J* = 2.0 Hz, 1H, arom H), 8.56 (dd, *J* = 4.7, 1.3 Hz, 1H, arom H), 8.75 (d, *J* = 1.9 Hz, 1H, arom H), 8.83 (d, *J* = 1.3 Hz, 1H, arom H), 12.44 (s, 1H, NH) ppm. ^{13}C NMR (150 MHz, DMSO) *δ:* 53.3 (OCH_3), 86.4 (C), 87.9 (C), 95.3 (C), 107.8 (CH), 115.5 (CH), 120.3 (C), 120.5 (C), 123.6 (CH), 125.9 (CH), 126.4 (C), 132.2 (CH), 138.2 (CH), 143.2 (CH), 147.5 (CH), 148.3 (C), 148.4 (CH), 148.8 (C), 151.5 (CH), 164.5 (C) ppm. HR-MS m/z $[M+H]^+$ calcd for $C_{20}H_{14}N_4O$ 327.1240, found 327.1240.

5-(3-Chlorophenyl)-3-(pyridin-3-ylethynyl)-1*H*-pyrrolo[2,3-*b*]pyridine (4h). This compound was obtained using the precursor **2a** and 3-chlorophenylboronic acid. The crude residue was purified by flash chromatography using a mixture of hexane and acetone (in a ratio of 7:3) as mobile phase, affording the title compound as a white solid in 77% yield (42.5 mg, 0.13 mmol). ^1H NMR (600 MHz, DMSO) *δ:* 7.43–7.48 (m, 2H, arom H), 7.53 (t, *J* = 7.9 Hz, 1H, arom H), 7.79 (ddd, *J* = 7.7, 1.7, 1.0 Hz, 1H, arom H), 7.89 (t, *J* = 1.9 Hz, 1H, arom H), 8.01–8.04 (m, 1H, arom H), 8.06 (s, 1H, arom H), 8.41 (d, *J* = 2.2 Hz, 1H, arom H), 8.56 (dd, *J* = 4.8, 1.7 Hz, 1H, arom H), 8.66 (d, *J* = 2.2 Hz, 1H, arom H), 8.83 (dd, *J* = 2.1, 0.8 Hz, 1H, arom H) ppm. ^{13}C NMR (150 MHz, DMSO) *δ:* 86.6 (C), 87.8 (C), 95.1 (C), 120.3 (C), 120.5 (C), 123.6 (CH), 125.7 (CH), 126.0 (CH), 126.9 (CH), 127.2 (CH), 128.1 (C), 130.9 (CH), 132.1 (CH), 133.9 (C), 138.2 (CH), 140.7 (C), 143.2 (CH), 147.7 (C), 148.3 (CH), 151.5 (CH) ppm. HR-MS m/z $[M+H]^+$ calcd for $C_{20}H_{12}ClN_3$ 330.0788, found 330.0792.

5-(4-Fluorophenyl)-3-(pyridin-3-ylethynyl)-1*H*-pyrrolo[2,3-*b*]pyridine (4i). This compound was obtained using the precursor **2a** and 4-fluorophenylboronic acid. The crude residue was purified by flash chromatography using a mixture of hexane and acetone (in a ratio of 7:3) as mobile phase, affording the title compound as a white solid in 78% yield (40.8 mg, 0.13 mmol). ^{1}H NMR (600 MHz, DMSO) δ: 7.29–7.36 (m, 2H, arom H), 7.45 (ddd, J = 7.9, 4.9, 0.9 Hz, 1H, arom H), 7.81–7.86 (m, 2H, arom H), 7.99–8.02 (m, 1H, arom H), 8.03 (s, 1H, arom H), 8.33 (d, J = 2.2 Hz, 1H, arom H), 8.55 (dd, J = 4.8, 1.6 Hz, 1H, arom H), 8.61 (d, J = 2.2 Hz, 1H, arom H), 8.81 (dd, J = 2.2, 0.9 Hz, 1H, arom H) ppm. ^{13}C NMR (150 MHz, DMSO) δ: 86.6 (C), 87.7 (C), 94.9 (C), 115.9 (d, J = 21.3 Hz, CH), 120.4 (d, J = 28.8 Hz, C), 123.6 (CH), 125.4 (CH), 128.6 (C), 129.3 (d, J = 8.1 Hz, CH), 131.9 (CH), 135.0 (C), 138.1 (CH), 143.1 (CH), 147.4 (C), 148.3 (CH), 151.4 (CH), 161.1 (C), 162.7 (C) ppm. ^{19}F NMR (471 MHz, DMSO) δ: -116.95 ppm. HR-MS m/z [M+H]$^+$ calcd for $C_{20}H_{12}FN_3$ 314.1088, found 314.1088.

5-(Furan-3-yl)-3-(pyridin-3-ylethynyl)-1*H*-pyrrolo[2,3-*b*]pyridine (4j). This compound was obtained using the precursor **3a** and 3-furanylboronic acid. The crude residue was purified by flash chromatography using a mixture of hexane and acetone (in a ratio of 3:2) as mobile phase, affording the title compound as a white solid in 73% yield (34.9 mg, 0.12 mmol). ^{1}H NMR (600 MHz, DMSO) δ: 7.15–7.18 (m, 1H, arom H), 7.46 (dd, J = 7.8, 4.9 Hz, 1H, arom H), 7.79 (t, J = 1.6 Hz, 1H, arom H), 7.98 (s, 1H, arom H), 7.99–8.03 (m, 1H, arom H), 8.30 (d, J = 2.0 Hz, 1H), 8.33 (s, 1H, arom H), 8.56 (dd, J = 4.8, 1.5 Hz, 1H, arom H), 8.65 (d, J = 2.0 Hz, 1H, arom H), 8.82 (d, J = 1.5 Hz, 1H, arom H), 12.27 (s, 1H, NH) ppm. ^{13}C NMR (150 MHz, DMSO) δ: 86.7 (C), 87.7 (C), 94.6 (C), 109.1 (CH), 120.3 (C), 120.5 (C), 121.5 (C), 123.7 (CH), 123.9 (C), 124.0 (CH), 131.6 (CH), 138.1 (CH), 139.2 (CH), 142.3 (CH), 144.4 (CH), 147.1 (C), 148.3 (CH), 151.5 (CH) ppm. HR-MS m/z [M+H]$^+$ calcd for $C_{18}H_{11}N_3O$ 286.0975, found 286.0975.

3-(Pyridin-3-ylethynyl)-5-(thiophen-3-yl)-1*H*-pyrrolo[2,3-*b*]pyridine (4k). This compound was obtained using the precursor **3a** and 3-thienylboronic acid. The crude residue was purified by flash chromatography using a mixture of hexane and acetone (in a ratio of 7:3) as mobile phase, affording the title compound as a beige solid in 79% yield (40.0 mg, 0.13 mmol). ^{1}H NMR (600 MHz, DMSO) δ: 7.46 (ddd, J = 7.9, 4.8, 0.8 Hz, 1H, arom H), 7.69 (dd, J = 5.0, 2.9 Hz, 1H), 7.74 (dd, J = 5.0, 1.3 Hz, 1H, arom H), 7.98– 8.04 (m, 3H, arom H), 8.39 (d, J = 2.1 Hz, 1H, arom H), 8.56 (dd, J = 4.8, 1.6 Hz, 1H, arom H), 8.74 (d, J = 2.1 Hz, 1H, arom H), 8.82 (dd, J = 2.0, 0.7 Hz, 1H, arom H), 12.29 (s, 1H, NH) ppm. ^{13}C NMR (150 MHz, DMSO) δ: 86.7 (C), 87.7 (C), 94.8 (C), 120.3 (C), 120.5 (C), 120.7 (CH), 123.6 (CH), 124.5 (CH), 124.8

(C), 126.6 (CH), 127.3 (CH), 131.7 (CH), 138.1 (CH), 139.5 (C), 142.9 (CH), 147.2 (C), 148.3 (CH), 151.4 (CH) ppm. HR-MS m/z $[M+H]^+$ calcd for $C_{18}H_{11}N_3S$ 302.0746, found 302.0754.

5-(3,5-Dimethoxyphenyl)-3-(pyridin-2-ylethynyl)-1*H*-pyrrolo[2,3-*b*]pyridine (5a). This compound was obtained using the precursor **3b** and 3,5-dimethoxyphenylboronic acid. The crude residue was purified by flash chromatography using a mixture of hexane and acetone (in a ratio of 3:2) as mobile phase and a second time using dichloromethane and ethyl acetate (7:3), affording the title compound as a white solid in 50% yield (29.8 mg, 0.08 mmol). ^{1}H NMR (600 MHz, DMSO) *δ:* 3.84 (s, 6H, 2 x OCH_3), 6.53 (t, *J* = 2.2 Hz, 1H, arom H), 6.90 (d, *J* = 2.2 Hz, 2H, arom H), 7.37 (ddd, *J* = 7.6, 4.9, 1.2 Hz, 1H), 7.70 (dt, *J* = 7.9, 1.0 Hz, 1H, arom H), 7.84 (td, *J* = 7.7, 1.8 Hz, 1H, arom H), 8.08 (s, 1H, arom H), 8.28 (d, *J* = 2.2 Hz, 1H, arom H), 8.59 (ddd, *J* = 4.8, 1.8, 1.0 Hz, 1H, arom H), 8.64 (d, *J* = 2.2 Hz, 1H, arom H) ppm. ^{13}C NMR (150 MHz, DMSO) *δ:* 55.5 (OCH_3), 83.2 (C), 91.0 (C), 94.5 (C), 99.4 (CH), 105.4 (CH), 120.2 (C), 122.9 (CH), 125.4 (CH), 127.0 (CH), 129.7 (C), 132.2 (CH), 132.6 (CH), 136.7 (CH), 140.7 (C), 143.3 (C), 143.4 (CH), 147.6 (C), 150.1 (CH), 161.0 (C) ppm. HR-MS m/z $[M+H]^+$ calcd for $C_{22}H_{17}N_3O_2$ 356.1393, found 356.1386.

5-(3,5-Dimethoxyphenyl)-3-(phenylethynyl)-1*H*-pyrrolo[2,3-*b*]pyridine (5b). This compound was obtained using the precursor **3c** and 3,5-dimethoxyphenylboronic acid. The crude residue was purified by flash chromatography using a mixture of hexane and acetone (in a ratio of 4:1) as mobile phase, affording the title compound as a light yellow solid in 80% yield (47.7 mg, 0.13 mmol). ^{1}H NMR (600 MHz, DMSO) *δ:* 3.85 (s, 6H, 2 x OCH_3), 6.54 (t, *J* = 2.2 Hz, 1H, arom H), 6.90 (d, *J* = 2.2 Hz, 2H, arom H), 7.36–7.46 (m, 3H, arom H), 7.60–7.64 (m, 2H, arom H), 7.99 (s, 1H, arom H), 8.29 (d, *J* = 2.2 Hz, 1H, arom H), 8.63 (d, *J* = 2.1 Hz, 1H, arom H), 12.27 (s, 1H, NH) ppm. ^{13}C NMR (150 MHz, DMSO) *δ:* 55.4 (OCH_3), 83.4 (C), 90.8 (C), 95.4 (C), 99.3 (CH), 105.4 (CH), 120.2 (C), 123.4 (C), 125.5 (CH), 128.1 (CH), 128.7 (CH), 129.5 (C), 131.1 (CH), 131.4 (CH), 140.8 (C), 143.2 (CH), 147.6 (C), 161.0 (C) ppm. HR-MS m/z $[M+H]^+$ calcd for $C_{23}H_{18}N_2O_2$ 355.1441, found 355.1432.

5-(3,5-Dimethoxyphenyl)-3-((4-fluorophenyl)ethynyl)-1*H*-pyrrolo[2,3-*b*]pyridine (5c). This compound was obtained using the precursor **3d** and 3,5-dimethoxyphenylboronic acid. The crude residue was purified by flash chromatography using a mixture of hexane and acetone (in a ratio of 4:1) as mobile phase and a second time using dichloromethane and ethyl acetate (4:1), affording the title compound as a light yellow solid in 78% yield (46.0 mg, 0.12 mmol). ^{1}H NMR (600 MHz, DMSO) *δ:* 3.83 (s, 6H, 2 x OCH_3), 6.52 (t, *J* = 2.2 Hz, 1H, arom H), 6.89

(d, J = 2.2 Hz, 2H, arom H), 7.22–7.29 (m, 2H, arom H), 7.63–7.69 (m, 2H, arom H), 7.96 (s, 1H, arom H), 8.29 (d, J = 2.2 Hz, 1H, arom H), 8.62 (d, J = 2.2 Hz, 1H, arom H), 12.25 (s, 1H, NH) ppm. ^{13}C NMR (150 MHz, DMSO) δ: 55.4 (OCH_3), 83.1 (C), 89.7 (C), 95.3 (C), 99.3 (CH), 105.4 (CH), 116.0 (d, J = 22.0 Hz, CH), 119.9 (d, J = 2.8 Hz, C), 120.2 (C), 125.5 (CH), 129.5 (C), 131.4 (CH), 133.4 (d, J = 8.4 Hz, CH), 140.8 (C), 143.3 (CH), 147.6 (C), 161.0 (C), 161.7 (d, J = 246.8 Hz, C) ppm. ^{19}F NMR (471 MHz, DMSO) δ: -112.77 ppm. HR-MS m/z $[M+H]^+$ calcd for $C_{23}H_{17}FN_2O_2$ 373.1347, found 373.1351.

3-((3-Chlorophenyl)ethynyl)-5-(3,5-dimethoxyphenyl)-1*H*-pyrrolo[2,3-*b*]pyridine (5d). This compound was obtained using the precursor **3e** and 3,5-dimethoxyphenylboronic acid. The crude residue was purified by flash chromatography using a mixture of hexane and acetone (in a ratio of 4:1) as mobile phase, affording the title compound as a white solid in 81% yield (47.4 mg, 0.12 mmol). ^{1}H NMR (600 MHz, DMSO) δ: 3.84 (s, 6H, 2 x OCH_3), 6.54 (t, J = 2.2 Hz, 1H, arom H), 6.90 (d, J = 2.3 Hz, 2H, arom H), 7.43–7.46 (m, 2H, arom H), 7.55–7.59 (m, 1H, arom H), 7.71 (dd, J = 2.6, 1.5 Hz, 1H, arom H), 8.01 (s, 1H, arom H), 8.34 (d, J = 2.1 Hz, 1H, arom H), 8.63 (d, J = 2.2 Hz, 1H, arom H), 12.32 (s, 1H, NH) ppm. ^{13}C NMR (150 MHz, DMSO) δ: 55.4 (OCH_3), 85.1 (C), 89.5 (C), 95.0 (C), 99.3 (CH), 105.5 (CH), 120.2 (C), 125.5 (C), 125.6 (CH), 128.1 (CH), 129.6 (C), 129.7 (CH), 130.5 (CH), 130.6 (CH), 131.9 (CH), 133.3 (C), 140.7 (C), 143.3 (CH), 147.6 (C), 161.0 (C) ppm. HR-MS m/z $[M+H]^+$ calcd for $C_{23}H_{17}ClN_2O_2$ 389.1051, found 389.1044.

5-(3,5-Dimethoxyphenyl)-3-((3-methoxyphenyl)ethynyl)-1*H*-pyrrolo[2,3-*b*]pyridine (5e). This compound was obtained using the precursor **3f** and 3,5-dimethoxyphenylboronic acid. The crude residue was purified by flash chromatography using a mixture of hexane and acetone (in a ratio of 4:1) as mobile phase, affording the title compound as a white solid in 83% yield (48.7 mg, 0.13 mmol). ^{1}H NMR (600 MHz, DMSO) δ: 3.80 (s, 3H, OCH_3), 3.83 (s, 6H, 2 x OCH_3), 6.52 (t, J = 2.2 Hz, 1H, arom H), 6.89 (d, J = 2.2 Hz, 2H, arom H), 6.95 (ddd, J = 8.3, 2.6, 0.9 Hz, 1H, arom H), 7.15 (dd, J = 2.5, 1.4 Hz, 1H, arom H), 7.16–7.20 (m, 1H, arom H), 7.30–7.35 (m, 1H, arom H), 7.97 (s, 1H, arom H), 8.28 (d, J = 2.1 Hz, 1H, arom H), 8.62 (d, J = 2.2 Hz, 1H, arom H), 12.26 (s, 1H, NH) ppm. ^{13}C NMR (150 MHz, DMSO) δ: 55.3 (OCH_3), 55.4 (OCH_3), 83.3 (C), 90.8 (C), 95.4 (C), 99.3 (CH), 105.4 (CH), 114.6 (CH), 115.9 (CH), 120.2 (C), 123.6 (CH), 124.5 (C), 125.5 (CH), 129.5 (C), 129.9 (CH), 131.5 (CH), 140.8 (C), 143.3 (CH), 147.6 (C), 159.3 (C), 161.0 (C) ppm. HR-MS m/z $[M+H]^+$ calcd for $C_{24}H_{20}N_2O_3$ 385.1547, found 385.1548.

5-(3,5-Dimethoxyphenyl)-3-(thiophen-3-ylethynyl)-1*H*-pyrrolo[2,3-*b*]pyridine (5f). This compound was obtained using the precursor **3g** and 3,5-dimethoxyphenylboronic acid. The crude residue was purified by flash chromatography using a mixture of hexane and acetone (in a ratio of 4:1) as mobile phase, affording the title compound as a white solid in 78% yield (46.3 mg, 0.13 mmol). ^{1}H NMR (600 MHz, DMSO) δ: 3.84 (s, 6H, 2 x OCH_3), 6.53 (t, *J* = 2.1 Hz, 1H, arom H), 6.89 (d, *J* = 2.2 Hz, 2H, arom H), 7.32 (dd, *J* = 4.9, 1.0 Hz, 1H, arom H), 7.64 (dd, *J* = 4.9, 3.0 Hz, 1H, arom H), 7.87 (dd, *J* = 2.9, 1.0 Hz, 1H, arom H), 7.95 (s, 1H, arom H), 8.24 (d, *J* = 2.1 Hz, 1H, arom H), 8.62 (d, *J* = 2.1 Hz, 1H, arom H) ppm. ^{13}C NMR (150 MHz, DMSO) δ: 55.4 (OCH_3), 82.4 (C), 86.1 (C), 95.4 (C), 99.3 (CH), 105.3 (CH), 120.2 (C), 122.2 (C), 125.4 (CH), 126.7 (CH), 128.8 (CH), 129.4 (C), 129.9 (CH), 131.2 (CH), 140.8 (C), 143.2 (CH) ppm, 147.6 (C), 161.0 (C). HR-MS m/z $[M+H]^+$ calcd for $C_{21}H_{16}N_2O_2S$ 361.1005, found 361.1000.

5-(3,5-Dimethoxyphenyl)-1-methyl-3-(pyridin-3-ylethynyl)-1*H*-pyrrolo[2,3-*b*]pyridine (7a). This compound was obtained using the precursor **6a** and 3,5-dimethoxyphenylboronic acid. The crude residue was purified by flash chromatography using a mixture of hexane and acetone (in a ratio of 7:3) as mobile phase and a second time using dichloromethane diethyl ether (9:1), affording the title compound as white foam in 76% yield (44.9 mg, 0.12 mmol). ^{1}H NMR (500 MHz, $CDCl_3$) δ: 3.88 (s, 6H, 2 x OCH_3), 3.95 (s, 3H, NCH_3), 6.50 (t, *J* = 2.2 Hz, 1H, arom H), 6.79 (d, *J* = 2.2 Hz, 2H, arom H), 7.29 (dd, *J* = 7.9, 4.9 Hz, 1H, aomr H), 7.53 (s, 1H, arom H), 7.83 (dt, *J* = 7.9, 1.8 Hz, 1H, arom H), 8.21 (d, *J* = 2.0 Hz, 1H, arom H), 8.53 (dd, *J* = 4.6, 1.0 Hz, 1H, arom H), 8.62 (d, *J* = 2.0 Hz, 1H, arom H), 8.80 (s, 1H, arom H) ppm. ^{13}C NMR (126 MHz, $CDCl_3$) δ: 31.6 (NCH_3), 55.5 (OCH_3), 85.7 (C), 88.0 (C), 95.3 (C), 99.0 (CH), 105.8 (CH), 121.0 (C), 123.0 (CH), 126.6 (CH), 130.5 (C), 133.5 (CH), 138.1 (CH), 141.2 (C), 143.5 (CH), 146.8 (C), 148.0 (CH), 151.9 (CH), 161.2 (C) ppm. HR-MS m/z $[M+H]^+$ calcd for $C_{23}H_{19}N_3O_2$ 370.1550, found 370.1545.

5-(3,5-Dimethoxyphenyl)-1-ethyl-3-(pyridin-3-ylethynyl)-1*H*-pyrrolo[2,3-*b*]pyridine (7b). This compound was obtained using the precursor **6b** and 3,5-dimethoxyphenylboronic acid. The crude residue was purified by flash chromatography using a mixture of hexane and acetone (in a ratio of 7:3) as mobile phase, affording the title compound as a white solid in 75% yield (44.1 mg, 0.11 mmol). ^{1}H NMR (600 MHz, DMSO) δ: 1.44 (t, *J* = 7.2 Hz, 3H, CH_3), 3.84 (s, 6H, 2 x OCH_3), 4.36 (q, *J* = 7.2 Hz, 2H, CH_2), 6.54 (t, *J* = 2.2 Hz, 1H, arom H), 6.90 (d, *J* = 2.2 Hz, 2H, arom H), 7.45 (ddd, *J* = 7.9, 4.8, 0.7 Hz, 1H, arom H), 7.99–8.03 (m, 1H, arom H), 8.15 (s, 1H, arom H), 8.34 (d, *J* = 2.1 Hz, 1H, arom H), 8.55 (dd, *J* = 4.8, 1.5 Hz, 1H, arom H),

8.66 (d, J = 2.1 Hz, 1H, arom H), 8.81 (d, J = 1.5 Hz, 1H, arom H) ppm. ^{13}C NMR (150 MHz, DMSO) δ: 15.5 (CH_3), 39.8 (CH_2, overlapped with DMSO signal, it was confirmed by HSQC), 55.4 (OCH_3), 86.4 (C), 88.0 (C), 94.0 (C), 99.3 (CH), 105.5 (CH), 120.5 (C), 120.6 (C), 123.6 (CH), 126.0 (CH), 129.8 (C), 134.0 (CH), 138.1 (CH), 140.5 (C), 143.1 (CH), 146.0 (C), 148.3 (CH), 151.4 (CH), 161.0 (C) ppm. HR-MS m/z $[M+H]^+$ calcd for $C_{24}H_{21}N_3O_2$ 384.1706, found 384.1705.

5-(3,5-Dimethoxyphenyl)-1-isobutyl-3-(pyridin-3-ylethynyl)-1*H*-pyrrolo[2,3-*b*]pyridine (7c). This compound was obtained using the precursor **6c** and 3,5-dimethoxyphenylboronic acid. The crude residue was purified by flash chromatography using a mixture of hexane and acetone (in a ratio of 4:1) as mobile phase and a second time using dichloromethane and ethyl acetate (9:1), affording the title compound as a white solid in 72% yield (41.8 mg, 0.10 mmol). ^{1}H NMR (600 MHz, $CDCl_3$) δ: 0.98 (d, J = 6.7 Hz, 6H, 2 x CH_3), 2.27–2.36 (m, 1H, CH), 3.88 (s, 6H, 2 x OCH_3), 4.14 (d, J = 7.4 Hz, 2H, CH_2), 6.50 (t, J = 2.2 Hz, 1H, arom H), 6.79 (d, J = 2.3 Hz, 2H, arom H), 7.29 (ddd, J = 7.7, 4.8, 0.8 Hz, 1H, arom H), 7.55 (s, 1H, arom H), 7.82–7.86 (m, 1H, arom H), 8.21 (d, J = 2.1 Hz, 1H, arom H), 8.54 (dd, J = 4.9, 1.6 Hz, 1H, arom H), 8.60 (d, J = 2.1 Hz, 1H, arom H), 8.80–8.81 (m, 1H, arom H) ppm. ^{13}C NMR (150 MHz, $CDCl_3$) δ: 20.1 (CH_3), 29.4 (CH), 52.5 (CH_2), 55.5 (OCH_3), 85.8 (C), 88.0 (C), 95.1 (C), 99.1 (CH), 105.9 (CH), 121.0 (C), 121.1 (C), 123.0 (CH), 126.6 (CH), 130.6 (C), 133.0 (CH), 138.1 (CH), 141.4 (C), 143.5 (CH), 146.8 (C), 148.1 (CH), 152.0 (CH), 161.2 (C) ppm. HR-MS m/z $[M+H]^+$ calcd for $C_{26}H_{25}N_3O_2$ 412.2019, found 412.2015.

1-Benzyl-5-(3,5-dimethoxyphenyl)-3-(pyridin-3-ylethynyl)-1H-pyrrolo[2,3-b]pyridine (7d). This compound was obtained using the precursor **6d** and 3,5-dimethoxyphenylboronic acid. The crude residue was purified by flash chromatography using a mixture of hexane and acetone (in a ratio of 4:1) as mobile phase and a second time using dichloromethane and ethyl acetate (9:1), affording the title compound as a white solid in 74% yield (42.5 mg, 0.09 mmol). ^{1}H NMR (600 MHz, $CDCl_3$) δ: 2.35 (s, 3H, OCH_3), 7.29–7.42 (m, 5H, arom H), 7.98 (dt, J = 7.9, 1.9 Hz, 1H, arom H), 8.08 (d, J = 2.0 Hz, 1H, arom H), 8.64 (dd, J = 4.9, 1.6 Hz, 1H, arom H), 8.89 (d, J = 2.0 Hz, 1H, arom H), 8.93 (d, J = 1.5 Hz, 1H, arom H) ppm. ^{13}C NMR (150 MHz, $CDCl_3$) δ: 20.4 (CH_3), 80.0 (C), 104.1 (C), 119.2 (C), 123.1 (CH), 126.4 (CH), 128.7 (CH), 128.8 (CH), 129.9 (CH), 130.8 (CH), 135.7 (C), 136.8 (C), 137.6 (C), 138.7 (CH), 143.4 (C), 147.6 (C), 149.8 (CH), 152.3 (CH), 153.8 (CH), 154.9 (C) ppm. HR-MS m/z $[M+H]^+$ calcd for $C_{29}H_{23}N_3O_2$ 328.0903, found 328.0912.

***N*-Methyl-4-(3-(pyridin-3-ylethynyl)-1*H*-pyrrolo[2,3-*b*]pyridin-5-yl)benzamide (8a).** This compound was obtained using the precursor **3a** and 4-(*N*-methylaminocarbonyl)phenylboronic acid. The crude residue was purified by flash chromatography using a mixture of hexane and acetone (in a ratio of 1:1) as mobile phase, affording the title compound as a white solid in 74% yield (43.7 mg, 0.12 mmol). ^{1}H NMR (600 MHz, DMSO) δ: 2.83 (d, *J* = 4.4 Hz, 1H, CH_3), 7.47 (dd, *J* = 7.7, 4.9 Hz, 1H, arom H), 7.92 (d, *J* = 8.3 Hz, 2H, arom H), 7.98 (d, *J* = 8.3 Hz, 2H, arom H), 8.02–8.04 (m, 1H, arom H), 8.06 (s, 1H, arom H), 8.43 (d, *J* = 2.0 Hz, 1H, arom H), 8.51–8.54 (m, 1H, CONH), 8.56 (dd, *J* = 4.8, 1.4 Hz, 1H, arom H), 8.71 (d, *J* = 2.0 Hz, 1H, arom H), 8.83 (d, *J* = 1.4 Hz, 1H, arom H), 12.38 (bs, 1H, NH) ppm. ^{13}C NMR (150 MHz, DMSO) δ: 26.4 (CH_3), 86.5 (C), 87.8 (C), 95.0 (C), 120.3 (C), 120.5 (C), 123.6 (CH), 125.6 (CH), 127.0 (CH), 127.9 (CH), 128.5 (C), 132.0 (CH), 133.1 (C), 138.2 (CH), 141.0 (C), 143.3 (CH), 147.7 (C), 148.3 (CH), 151.5 (CH), 166.3 (C) ppm. HR-MS m/z $[M+H]^+$ calcd for $C_{22}H_{16}N_4O$ 353.1397, found 353.1392.

***N*-Ethyl-4-(3-(pyridin-3-ylethynyl)-1*H*-pyrrolo[2,3-*b*]pyridin-5-yl)benzamide (8b).** This compound was obtained using the precursor **3a** and 4-(*N*-ethylaminocarbonyl)phenylboronic acid. The crude residue was purified by flash chromatography using a mixture of hexane and acetone (in a ratio of 3:2) as mobile phase, affording the title compound as a white solid in 76% yield (46.7 mg, 0.13 mmol). ^{1}H NMR (600 MHz, DMSO) δ: 1.15 (t, *J* = 7.2 Hz, 3H, CH_3), 3.29–3.35 (m, 2H, CH_2), 7.46 (dd, *J* = 7.8, 4.9 Hz, 1H, arom H), 7.91 (d, *J* = 8.4 Hz, 2H, arom H), 7.97 (d, *J* = 8.4 Hz, 2H, arom H), 8.00–8.03 (m, 1H, arom H), 8.05 (s, 1H, arom H), 8.42 (d, *J* = 2.1 Hz, 1H, arom H), 8.51–8.58 (m, 2H, CONH, arom H), 8.70 (d, *J* = 2.1 Hz, 1H, arom H), 8.82 (d, *J* = 1.5 Hz, 1H, arom H), 12.38 (s, 1H, NH) ppm. ^{13}C NMR (150 MHz, DMSO) δ: 14.9 (CH_3), 34.2 (CH_2), 86.6 (C), 87.8 (C), 95.0 (C), 120.3 (C), 120.5 (C), 123.7 (CH), 125.6 (CH), 126.9 (CH), 127.9 (CH), 128.6 (C), 132.0 (CH), 133.3 (C), 138.2 (CH), 140.9 (C), 143.3 (CH), 147.7 (C), 148.4 (CH), 151.5 (CH), 165.6 (C) ppm. HR-MS m/z $[M+H]^+$ calcd for $C_{23}H_{18}N_4O$ 367.1553, found 367.1547.

***N*,*N*-Diethyl-3-(3-(pyridin-3-ylethynyl)-1*H*-pyrrolo[2,3-*b*]pyridin-5-yl)benzamide (8c).** This compound was obtained using the precursor **2a** and 4-(*N*,*N*-diethylaminocarbonyl)phenylboronic acid pinacol ester. The crude residue was purified by flash chromatography using a mixture of hexane and acetone (in a ratio of 3:2) as mobile phase, affording the title compound as a white solid in 77% yield (51.6 mg, 0.13 mmol). ^{1}H NMR (600 MHz, DMSO) δ: 1.04–1.20 (m, 6H, 2 x CH_3), 3.26 (bs, 2H, CH_2), 3.45 (bs, 2H, CH_2), 7.43–7.49 (m, 3H, arom H), 7.87 (d, *J* = 8.2 Hz, 2H, arom H), 8.00–8.03 (m, 1H, arom H), 8.04 (d, *J*

= 8.1 Hz, 1H, arom H), 8.40 (d, *J* = 2.1 Hz, 1H, arom H), 8.55 (dd, *J* = 4.8, 1.5 Hz, 1H, arom H), 8.68 (d, *J* = 2.1 Hz, 1H, arom H), 8.82 (d, *J* = 1.5 Hz, 1H, arom H), 12.36 (s, 1H, NH) ppm. ^{13}C NMR (150 MHz, DMSO) *δ:* 12.9 (CH_3), 14.2 (CH_3), 38.8 (CH_2), 43.0 (CH_2), 86.6 (C), 87.8 (C), 95.0 (C), 120.4 (C), 120.5 (C), 125.5 (CH), 123.6 (CH), 127.0 (CH), 127.1 (CH), 128.7 (C), 131.9 (CH), 136.1 (C), 138.1 (CH), 139.1 (C), 143.2 (CH), 147.6 (C), 148.3 (CH), 151.4 (CH), 169.8 (C) ppm. HR-MS m/z $[M+H]^+$ calcd for $C_{25}H_{22}N_4O$ 395.1866, found 395.1859.

***N*-Cyclopropyl-4-(3-(pyridin-3-ylethynyl)-1*H*-pyrrolo[2,3-*b*]pyridin-5-yl)benzamide (8d).** This compound was obtained using the precursor **2a** and 4-(*N*-cyclopropylaminocarbonyl)phenylboronic acid. The crude residue was purified by flash chromatography using a mixture of hexane and acetone (in a ratio of 3:2) as mobile phase, affording the title compound as a white solid in 79% yield (50.1 mg, 0.13 mmol). ^{1}H NMR (600 MHz, DMSO) *δ:* 0.58–0.63 (m, 2H, CH_2), 0.69–0.74 (m, 2H, CH_2), 2.85–2.91 (m, 1H, CH), 7.46 (ddd, *J* = 7.9, 4.8, 0.6 Hz, 1H, arom H), 7.90 (d, *J* = 8.4 Hz, 2H, arom H), 7.95 (d, *J* = 8.4 Hz, 2H, arom H), 8.01 (dt, *J* = 7.9, 1.9 Hz, 1H, arom H), 8.05 (s, 1H, arom H), 8.41 (d, *J* = 2.2 Hz, 1H, arom H), 8.51 (d, *J* = 4.1 Hz, 1H, NH), 8.55 (dd, *J* = 4.8, 1.6 Hz, 1H, arom H), 8.70 (d, *J* = 2.1 Hz, 1H, arom H), 8.82 (d, *J* = 1.5 Hz, 1H, arom H), 12.31 (bs, 1H, NH) ppm. ^{13}C NMR (150 MHz, DMSO) *δ:* 5.9 (CH_2), 23.2 (CH), 86.6 (C), 87.8 (C), 95.0 (C), 120.3 (C), 120.5 (C), 123.6 (CH), 125.6 (CH), 126.9 (CH), 128.0 (CH), 128.5 (C), 132.0 (CH), 133.0 (C), 138.2 (CH), 141.0 (C), 143.3 (CH), 147.7 (C), 148.4 (CH), 151.5 (CH), 167.2 (C) ppm. HR-MS m/z $[M+H]^+$ calcd for $C_{24}H_{18}N_4O$ 379.1553, found 379.1544.

***N*-Methyl-3-(3-(pyridin-3-ylethynyl)-1*H*-pyrrolo[2,3-*b*]pyridin-5-yl)benzamide (8e).** This compound was obtained using the precursor **2a** and 3-(*N*-methylaminocarbonyl)phenylboronic acid. The crude residue was purified by flash chromatography using a mixture of dichloromethane and acetone (in a ratio of 1:1) as mobile phase, affording the title compound as a white solid in 67% yield (39.6 mg, 0.11 mmol). ^{1}H NMR (600 MHz, DMSO) *δ*: 2.83 (d, *J* = 4.5 Hz, 3H, CH_3), 7.46 (ddd, *J* = 7.9, 4.9, 0.8 Hz, 1H, arom H), 7.59 (t, *J* = 7.7 Hz, 1H, arom H), 7.83–7.87 (m, 1H, arom H), 7.92–7.96 (m, 1H, arom H), 7.99–8.03 (m, 1H, arom H), 8.05 (s, 1H, arom H), 8.22 (t, *J* = 1.6 Hz, 1H, arom H), 8.41 (d, *J* = 2.2 Hz, 1H, arom H), 8.55 (dd, *J* = 4.8, 1.6 Hz, 1H, arom H), 8.58–8.61 (m, 1H, CONH), 8.70 (d, *J* = 2.2 Hz, 1H, arom H), 8.81 (dd, *J* = 2.2, 0.8 Hz, 1H, arom H) ppm.^{13}C NMR (150 MHz, DMSO) *δ*: 26.4 (CH_3), 86.6 (C), 87.7 (C), 94.9 (C), 120.3 (C), 120.5 (C), 123.7 (CH), 125.6 (CH), 126.7 (CH), 129.0 (C), 129.2 (CH), 129.8 (CH), 132.1 (CH), 135.3 (C), 138.2 (CH), 138.5 (C), 143.3 (CH), 147.6 (C), 148.3

(CH), 151.5 (CH), 166.6 (C) ppm. HR-MS m/z $[M+H]^+$ calcd for $C_{22}H_{16}N_4O$ 353.1397, found 353.1383.

***N*-Cyclopropyl-3-(3-(pyridin-3-ylethynyl)-1*H*-pyrrolo[2,3-*b*]pyridin-5-yl)benzamide (8f).** This compound was obtained using the precursor **3a** and 3-(*N*-cyclopropylaminocarbonyl)phenylboronic acid pinacol ester. The crude residue was purified by flash chromatography using a mixture of dichloromethane and methanol (in a ratio of 10:0.3) as mobile phase, affording the title compound as a white solid in 78% yield (50.2 mg, 0.13 mmol). ^{1}H NMR (600 MHz, DMSO) δ: 0.58–0.63 (m, 2H, CH_2), 0.69–0.75 (m, 2H, CH_2), 2.85–2.92 (m, 1H, CH), 7.46 (ddd, J = 7.9, 4.9, 0.8 Hz, 1H, arom H), 7.58 (t, J = 7.7 Hz, 1H, arom H), 7.82–7.86 (m, 1H, arom H), 7.92–7.96 (m, 1H, arom H), 8.00–8.03 (m, 1H, arom H), 8.05 (s, 1H, arom H), 8.18–8.19 (m, 1H, arom H), 8.40 (d, J = 2.1 Hz, 1H, arom H), 8.55 (dd, J = 4.8, 1.6 Hz, 1H, arom H), 8.58 (d, J = 4.0 Hz, 1H, NH), 8.70 (d, J = 2.2 Hz, 1H, arom H), 8.82 (dd, J = 2.1, 0.7 Hz, 1H, arom H), 12.37 (s, 1H, NH) ppm. ^{13}C NMR (150 MHz, DMSO) δ: 5.9 (CH_2), 23.2 (CH), 86.6 (C), 87.7 (C), 94.9 (C), 120.3 (C), 120.5 (C), 123.6 (CH), 125.6 (CH), 125.7 (CH), 126.3 (CH), 129.0 (C), 129.1 (CH), 129.9 (CH), 132.0 (CH), 135.2 (C), 138.2 (CH), 138.5 (C), 143.4 (CH), 147.6 (C), 148.4 (CH), 151.4 (CH), 167.4 (C) ppm. HR-MS m/z $[M+H]^+$ calcd for $C_{24}H_{18}N_4O$ 379.1553, found 379.1551.

***N*-Isobutyl-3-(3-(pyridin-3-ylethynyl)-1*H*-pyrrolo[2,3-*b*]pyridin-5-yl)benzamide (8g).** This compound was obtained using the precursor **3a** and 3-(*N*-isobutylaminocarbonyl)phenylboronic acid. The crude residue was purified by flash chromatography using a mixture of hexane and acetone (in a ratio of 3:2) as mobile phase, affording the title compound as a white solid in 71% yield (46.9 mg, 0.12 mmol). ^{1}H NMR (600 MHz, DMSO) δ: 0.91 (s, 3H, CH_3), 0.92 (s, 3H, CH_3), 1.84–1.92 (m, 1H, CH), 3.11–3.15 (m, 2H, CH_2), 7.45 (ddd, J = 7.9, 4.9, 0.7 Hz, 1H, arom H), 7.59 (t, J = 7.7 Hz, 1H, arom H), 7.87 (d, J = 7.8 Hz, 1H, arom H), 7.95 (d, J = 7.9 Hz, 1H, arom H), 8.01 (dt, J = 7.9, 1.9 Hz, 1H, arom H), 8.05 (s, 1H, arom H), 8.23 (s, 1H, arom H), 8.41 (d, J = 2.2 Hz, 1H, arom H), 8.56 (dd, J = 4.8, 1.6 Hz, 1H, arom H), 8.63 (t, J = 5.7 Hz, 1H, CONH), 8.72 (d, J = 2.1 Hz, 1H, arom H), 8.81 (d, J = 1.5 Hz, 1H, arom H) ppm. ^{13}C NMR (150 MHz, DMSO) δ: 20.4 (CH_3), 28.3 (CH), 46.9 (CH_2), 86.6 (C), 87.8 (C), 95.0 (C), 120.3 (C), 120.5 (C), 123.7 (CH), 125.6 (CH), 125.7 (CH), 126.4 (CH), 129.0 (C), 129.2 (CH), 129.8 (CH), 132.0 (CH), 135.6 (C), 138.2 (CH), 138.5 (C), 143.4 (CH), 147.6 (C), 148.4 (CH), 151.4 (CH), 166.2 (C) ppm. HR-MS m/z $[M+H]^+$ calcd for $C_{25}H_{22}N_4O$ 395.1866, found 395.1848.

***N*-(2-Methoxyethyl)-3-(3-(pyridin-3-ylethynyl)-1*H*-pyrrolo[2,3-*b*]pyridin-5-yl)benzamide (8h).** This compound was obtained using the precursor **2a** and 3-(2-

methoxyethylaminocarbonyl)benzeneboronic acid pinacol ester. The crude residue was purified by flash chromatography using a mixture of hexane and acetone (in a ratio of 3:2) as mobile phase, affording the title compound as a white solid in 69% yield (45.8 mg, 0.12 mmol). ^{1}H NMR (600 MHz, DMSO) δ: 3.28 (s, 3H, OCH_3), 3.45–3.50 (m, 4H, 2 x CH_2), 7.45 (ddd, J = 7.9, 4.8, 0.9 Hz, 1H, arom H), 7.59 (t, J = 7.7 Hz, 1H, arom H), 7.85–7.88 (m, 1H, arom H), 7.96 (ddd, J = 7.7, 1.7, 1.1 Hz, 1H, arom H), 8.00–8.03 (m, 1H, arom H), 8.05 (s, 1H, arom H), 8.24 (t, J = 1.7 Hz, 1H, NH), 8.41 (d, J = 2.2 Hz, 1H, arom H), 8.55 (dd, J = 4.8, 1.7 Hz, 1H, arom H), 8.69–8.72 (m, 2H, arom H), 8.81 (dd, J = 2.2, 0.9 Hz, 1H, arom H) ppm. ^{13}C NMR (150 MHz, DMSO) δ: 58.1 (OCH_3), 70.6 (CH_2), 86.6 (C), 87.8 (C), 95.0 (C), 120.3 (C), 120.5 (C), 123.7 (CH), 125.6 (CH), 125.7 (CH), 126.4 (CH), 129.0 (C), 129.2 (CH), 130.0 (CH), 132.1 (CH), 135.2 (C), 138.2 (CH), 138.5 (C), 143.4 (CH), 147.6 (C), 148.4 (CH), 151.5 (CH), 166.3 (C) ppm. HR-MS m/z $[M+H]^+$ calcd for $C_{24}H_{20}N_4O_2$ 397.1659, found 397.1651.

***N*-Benzyl-3-(3-(pyridin-3-ylethynyl)-1*H*-pyrrolo[2,3-*b*]pyridin-5-yl)benzamide (8i).** This compound was obtained using the precursor **3a** and 3-(benzylaminocarbonyl)benzeneboronic acid pinacol ester. The crude residue was purified by flash chromatography using a mixture of hexane and acetone (in a ratio of 3:2) as mobile phase, affording the title compound as a beige solid in 73% yield (52.4 mg, 0.12 mmol). ^{1}H NMR (600 MHz, DMSO) δ: 4.54 (d, J = 5.9 Hz, 2H, CH_2), 7.22–7.26 (m, 1H, arom H), 7.31–7.37 (m, 4H, arom H), 7.45 (ddd, J = 7.9, 4.9, 0.7 Hz, 1H, arom H), 7.61 (t, J = 7.7 Hz, 1H, arom H), 7.92 (d, J = 7.8 Hz, 1H, arom H), 7.98 (d, J = 7.7 Hz, 1H, arom H), 7.99–8.02 (m, 1H, arom H), 8.05 (s, 1H, arom H), 8.30 (bs, 1H, arom H), 8.42 (d, J = 2.1 Hz, 1H, arom H), 8.55 (dd, J = 4.8, 1.6 Hz, 1H, arom H), 8.72 (d, J = 2.1 Hz, 1H, arom H), 8.81 (d, J = 1.5 Hz, 1H, arom H), 9.21 (t, J = 5.9 Hz, 1H, NH), 12.37 (s, 1H, NH) ppm. ^{13}C NMR (150 MHz, DMSO) δ: 42.8 (CH_2), 86.6 (C), 87.8 (C), 95.0 (C), 120.3 (C), 120.5 (C), 123.7 (CH), 125.6 (CH), 125.8 (CH), 126.5 (CH), 126.9 (CH), 127.4 (CH), 128.4 (CH), 129.0 (C), 129.3 (CH), 130.1 (CH), 132.1 (CH), 135.1 (C), 138.2 (CH), 138.6 (C), 139.7 (C), 143.4 (CH), 147.6 (C), 148.4 (CH), 151.5 (CH), 166.2 (C) ppm. HR-MS m/z $[M+H]^+$ calcd for $C_{28}H_{20}N_4O$ 429.1710, found 429.1703.

***N*,*N*-Dimethyl-3-(3-(pyridin-3-ylethynyl)-1*H*-pyrrolo[2,3-*b*]pyridin-5-yl)benzamide (8j).** This compound was obtained using the precursor **3a** and 3-(*N*,*N*-dimethylaminocarbonyl)phenylboronic acid. The crude residue was purified by flash chromatography using a mixture of hexane and acetone (in a ratio of 3:2) as mobile phase, affording the title compound as a white solid in 75% yield (46.0 mg, 0.12 mmol). ^{1}H NMR (600 MHz, DMSO) δ: 2.97 (s, 1H, CH_3), 3.02 (s, 1H, CH_3), 7.38–7.42 (m, 1H, arom H), 7.45 (ddd,

J = 7.9, 4.9, 0.8 Hz, 1H, arom H), 7.56 (t, *J* = 7.7 Hz, 1H, arom H), 7.79 (t, *J* = 1.6 Hz, 1H, arom H), 7.87 (ddd, *J* = 7.8, 1.8, 1.1 Hz, 1H, arom H), 7.98–8.03 (m, 1H, arom H), 8.04 (s, 1H, arom H), 8.38 (d, *J* = 2.2 Hz, 1H, arom H), 8.55 (dd, *J* = 4.8, 1.6 Hz, 1H, arom H), 8.65 (d, *J* = 2.2 Hz, 1H, arom H), 8.81 (dd, *J* = 2.2, 0.8 Hz, 1H, arom H) ppm. ^{13}C NMR (150 MHz, DMSO) *δ:* 34.8 (CH_3), 86.6 (C), 87.7 (C), 95.0 (C), 120.3 (C), 120.5 (C), 123.6 (CH), 125.5 (CH), 125.6 (CH), 125.8 (CH), 128.1 (CH), 128.9 (C), 129.1 (CH), 132.0 (CH), 137.5 (C), 138.2 (CH), 138.6 (C), 143.3 (CH), 147.6 (C), 148.3 (CH), 151.5 (CH), 170.1 (C) ppm. HR-MS m/z $[M+H]^+$ calcd for $C_{23}H_{18}N_4O$ 367.1553, found 367.1547.

***N*,*N*-Diethyl-3-(3-(pyridin-3-ylethynyl)-1*H*-pyrrolo[2,3-*b*]pyridin-5-yl)benzamide (8k).** This compound was obtained using the precursor **3a** and 3-(*N*,*N*-diethylaminocarbonyl)phenylboronic acid. The crude residue was purified by flash chromatography using a mixture of hexane and acetone (in a ratio of 7:3) as mobile phase, affording the title compound as a white solid in 76% yield (50.3 mg, 0.13 mmol). ^{1}H NMR (600 MHz, $CDCl_3$) *δ:* 1.17 (bs, 3H, CH_3), 1.30 (bs, 3H, CH_3), 3.35 (bs, 2H, NCH_2), 3.61 (bs, 2H, NCH_2), 7.30 (dd, *J* = 7.7, 4.9 Hz, 1H, arom H), 7.39 (d, *J* = 7.6 Hz, 1H, arom H), 7.52 (t, *J* = 7.6 Hz, 1H, arom H), 7.68-7.72 (m, 2H, arom H), 7.74 (s, 1H, arom H), 7.86 (dt, *J* = 7.8, 1.7 Hz, 1H, arom H), 8.29 (d, *J* = 1.9 Hz, 1H, arom H), 8.55 (d, *J* = 3.9 Hz, 1H, arom H), 8.62 (d, *J* = 1.6 Hz, 1H, arom H), 8.82 (s, 1H, arom H), 11.68 (d, *J* = 11.5 Hz, 1H, NH) ppm. ^{13}C NMR (150 MHz, $CDCl_3$) *δ:* 12.9 (CH_3), 14.3 (CH_3), 39.4 (NCH_2), 43.4 (NCH_2), 85.7 (C), 87.8 (C), 96.7 (C), 121.0 (C), 121.1 (C), 123.0 (CH), 124.9 (CH), 125.5 (CH), 127.0 (CH), 128.2 (CH), 129.0 (CH), 129.9 (C), 130.3 (CH), 137.9 (C), 138.2 (CH), 139.4 (C), 143.1 (CH), 147.6 (C), 148.1 (CH), 152,0 (CH), 171.2 (C) ppm. HR-MS m/z $[M+H]^+$ calcd for $C_{25}H_{22}N_4O$ 395.1866, found 395.1867.

(3-(3-(Pyridin-3-ylethynyl)-1*H*-pyrrolo[2,3-*b*]pyridin-5-yl)phenyl)(pyrrolidin-1-yl)methanone (8l). This compound was obtained using the precursor **3a** and 3-(pyrrolidine-1-carbonyl)benzeneboronic acid pinacol ester. The crude residue was purified by flash chromatography using a mixture of hexane and acetone (in a ratio of 3:2) as mobile phase, affording the title compound as a white solid in 73% yield (48.0 mg, 0.12 mmol). ^{1}H NMR (600 MHz, $CDCl_3$) *δ:* 1.89–1.95 (m, 2H, CH_2), 1.96–2.04 (m, 2H, CH_2), 3.52 (t, *J* = 6.6 Hz, 2H, NCH_2), 3.72 (t, *J* = 7.0 Hz, 2H, NCH_2), 7.30 (ddd, *J* = 7.9, 5.0, 0.5 Hz, 1H, arom H), 7.50–7.54 (m, 2H, arom H), 7.70 (d, *J* = 1.5 Hz, 1H, arom H), 7.71–7.74 (m, 1H, arom H), 7.86 (dt, *J* = 7.9, 1.9 Hz, 1H, arom H), 7.91 (s, 1H, arom H), 8.29 (d, *J* = 2.0 Hz, 1H, arom H), 8.54 (dd, *J* = 4.8, 1.4 Hz, 1H, arom H), 8.63 (d, *J* = 1.8 Hz, 1H, arom H), 8.81 (d, *J* = 1.4 Hz, 1H, arom H), 11.28 (s, 1H, NH) ppm. ^{13}C NMR (150 MHz, $CDCl_3$) *δ:* 24.5 (CH_2), 26.4 (CH_2), 46.3 (NCH_2), 49.7 (NCH_2), 85.7 (C), 87.8 (C), 96.8 (C), 121.0 (C), 121.0 (C), 123.0 (CH), 125.7 (CH), 126.4 (CH), 126.9 (CH), 128.8 (CH), 128.9

(CH), 129.9 (C), 130.2 (CH), 137.9 (C), 138.2 (CH), 139.3 (C), 143.3 (CH), 147.5 (C), 148.1 (CH), 152.0 (CH), 170.0 (C) ppm. HR-MS m/z $[M+H]^+$ calcd for $C_{25}H_{20}N_4O$ 393.1710, found 393.1697.

Morpholino(3-(3-(pyridin-3-ylethynyl)-1*H*-pyrrolo[2,3-*b*]pyridin-5-yl)phenyl)methanone (8m). This compound was obtained using the precursor **3a** and 3-(4-morpholinylcarbonyl)benzeneboronic acid pinacol ester. The crude residue was purified by flash chromatography using a mixture of hexane and acetone (in a ratio of 3:2) as mobile phase, affording the title compound as a beige solid in 70% yield (47.9 mg, 0.12 mmol). ^{1}H NMR (600 MHz, $CDCl_3$) *δ:* 3.54 (bs, 2H), 3.67 (bs, 2H), 3.75–3.90 (m, 4H), 7.31 (dd, *J* = 7.8, 4.9 Hz, 1H, arom H), 7.41 (d, *J* = 7.6 Hz, 1H, arom H), 7.55 (t, *J* = 7.7 Hz, 1H, arom H), 7.71 (d, *J* = 1.9 Hz, 1H, arom H), 7.73–7.76 (m, 1H, arom H), 7.78 (bs, 1H, arom H), 7.86 (dt, *J* = 7.9, 1.8 Hz, 1H, arom H), 8.30 (d, *J* = 2.0 Hz, 1H, arom H), 8.55 (dd, *J* = 4.8, 1.5 Hz, 1H, arom H), 8.63 (d, *J* = 2.0 Hz, 1H, arom H), 8.82 (d, *J* = 1.4 Hz, 1H, arom H), 11.12 (s, 1H, NH) ppm. ^{13}C NMR (150 MHz, $CDCl_3$) *δ:* 66.9 (CH_2), 85.5 (C), 87.9 (C), 97.0 (C), 120.9 (C), 121.0 (C), 123.1 (CH), 125.7 (CH), 126.3 (CH), 127.0 (CH), 128.9 (CH), 129.2 (CH), 129.8 (C), 130.1 (CH), 136.1 (C), 138.2 (CH), 139.7 (C), 143.2 (CH), 147.6 (C), 148.2 (CH), 152.0 (CH), 170.3 (C) ppm. HR-MS m/z $[M+H]^+$ calcd for $C_{25}H_{20}N_4O_2$ 409.1659, found 409.1660.

***N*-Cyclopropyl-3-(3-((6-fluoropyridin-3-yl)ethynyl)-1*H*-pyrrolo[2,3-*b*]pyridin-5-yl)benzamide (9a)**. This compound was obtained using the precursor **3i** and 3-(*N*-cyclopropylaminocarbonyl)phenylboronic acid pinacol ester. The crude residue was purified by flash chromatography using a mixture of hexane and acetone (in a ratio of 7:3) as mobile phase, affording the title compound as a white solid in 77% yield (48.2 mg, 0.12 mmol). ^{1}H NMR (300 MHz, DMSO) *δ*: 0.58–0.66 (m, 2H, CH_2). 0.68–0.78 (m, 2H, CH_2), 2.89 (td, *J* = 7.2, 3.8 Hz, 1H, CH), 7.29 (dd, *J* = 8.5, 2.7 Hz, 1H, arom H), 7.59 (t, *J* = 7.7 Hz, 1H, arom H), 7.85 (d, *J* = 7.7 Hz, 1H, arom H), 7.95 (d, *J* = 7.7 Hz, 1H, arom H), 8.05 (s, 1H, arom H), 8.18–8.27 (m, 2H, arom H), 8.41 (d, *J* = 2.0 Hz, 1H, arom H), 8.52 (s, 1H, arom H), 8.59 (d, *J* = 3.8 Hz, 1H, CONH), 8.71 (d, *J* = 1.9 Hz, 1H, arom H), 12.38 (s, 1H, NH) ppm. ^{19}F NMR (282 MHz, DMSO) *δ:* -67.61 ppm. ^{13}C NMR (75 MHz, DMSO) *δ*: 6.2 (CH_2), 23.7 (CH), 86.8 (C), 95.2 (C), 110.3 (d, *J* = 38.3 Hz, CH), 119.2 (C), 120.7 (C), 126.0 (CH), 126.0 (CH), 126.7 (CH), 129.4 (C), 129.5 (CH), 130.3 (CH), 132.4 (CH), 135.6 (C), 138.9 (C), 143.7 (CH), 144.6 (d, *J* = 8.4 Hz, CH), 148.0 (C), 150.2 (d, *J* = 15.4 Hz, CH), 162.29 (d, *J* = 238.4 Hz, CF), 167.8 (C) ppm. HR-MS m/z $[M+H]^+$ calcd for $C_{24}H_{17}FN_4O$ 397.1459, found 397.1446.

***N*-Cyclopropyl-3-(3-((5-methoxypyridin-3-yl)ethynyl)-1*H*-pyrrolo[2,3-*b*]pyridin-5-yl)benzamide (9b)**. This compound was obtained using the precursor **3j** and 3-(*N*-

cyclopropylaminocarbonyl)phenylboronic acid pinacol ester. The crude residue was purified by flash chromatography using a mixture of hexane and acetone (in a ratio of 3:2) as mobile phase, affording the title compound as a white solid in 81% yield (50.4 mg, 0.12 mmol). ^{1}H NMR (300 MHz, DMSO) *δ:* 0.56–0.65 (m, 2H, CH_2), 0.68–0.79 (m, 2H, CH_2), 2.84–2.94 (m, 1H, CH), 3.89 (s, 3H, OCH_3), 7.55–7.65 (m, 2H, arom H), 7.85 (d, *J* = 7.4 Hz, 1H, arom H), 7.95 (d, *J* = 7.4 Hz, 1H, arom H), 8.06 (s, 1H, arom H), 8.20 (s, 1H, arom H), 8.29 (d, *J* = 2.0 Hz, 1H, arom H), 8.39–8.44 (m, 2H, arom H), 8.59 (d, *J* = 2.7 Hz, 1H, CONH), 8.71 (s, 1H, arom H), 12.39 (s, 1H, NH) ppm. ^{13}C NMR (75 MHz, DMSO) *δ:* 6.2 (CH_2), 23.6 (CH), 56.2 (OCH_3), 86.9 (C), 88.1 (C), 95.3 (C), 120.7 (C), 121.2 (C), 122.1 (CH), 126.0 (CH), 126.7 (CH), 129.4 (C), 129.5 (CH), 130.3 (CH), 132.5 (CH), 135.6 (C), 137.5 (CH), 138.8 (C), 143.7 (CH), 144.0 (CH), 148.0 (C), 155.5 (C), 167.8 (C) ppm. HR-MS m/z $[M+H]^+$ calcd for $C_{25}H_{20}N_4O_2$ 409.1659, found 409.1659.

***N*-Cyclopropyl-3-(3-((5-methylpyridin-3-yl)ethynyl)-1*H*-pyrrolo[2,3-*b*]pyridin-5-yl)benzamide (9c)**. This compound was obtained using the precursor **3h** and 3-(*N*-cyclopropylaminocarbonyl)phenylboronic acid pinacol ester. The crude residue was purified by flash chromatography using a mixture of hexane and acetone (in a ratio of 7:3) as mobile phase, affording the title compound as a white solid in 79% yield (49.6 mg, 0.13 mmol). ^{1}H NMR (300 MHz, DMSO) *δ*: 0.57–0.66 (m, 2H, CH_2), 0.68–0.78 (m, 2H, CH_2), 2.34 (s, 3H, CH_3), 2.84–2.95 (m, 1H, CH), 7.59 (t, *J* = 7.7 Hz, 1H, arom H), 7.81–7.88 (m, 2H, arom H), 7.95 (d, *J* = 7.5 Hz, 1H, arom H), 8.04 (s, 1H, arom H), 8.20 (s, 1H, arom H), 8.40 (bs, 2H, arom H), 8.57–8.63 (m, 2H, arom H and CONH), 8.71 (s, 1H, arom H), 12.36 (s, 1H, NH) ppm. ^{13}C NMR (75 MHz, DMSO) *δ*: 6.2 (CH_2), 18.1 (CH_3), 23.6 (CH), 86.7 (C), 88.2 (C), 95.4 (C), 120.3 (C), 120.7 (C), 125.9 (CH), 126.0 (CH), 126.7 (CH), 129.4 (C), 129.5 (CH), 130.3 (CH), 132.3 (CH), 133.4 (C), 135.6 (C), 138.7 (CH), 138.8 (C), 143.7 (CH), 148.0 (C), 148.9 (CH), 149.2 (CH), 167.8 (C) ppm. HR-MS m/z $[M+H]^+$ calcd for $C_{25}H_{20}N_4O$ 393.1710, found 393.1700.

***N*-Cyclopropyl-3-(3-(phenylethynyl)-1*H*-pyrrolo[2,3-*b*]pyridin-5-yl)benzamide (9d)**. This compound was obtained using the precursor **3c** and 3-(*N*-cyclopropylaminocarbonyl)phenylboronic acid pinacol ester. The crude residue was purified by flash chromatography using a mixture of hexane and acetone (in a ratio of 7:3) as mobile phase, affording the title compound as a light yellow solid in 81% yield (51.4 mg, 0.13 mmol). ^{1}H NMR (300 MHz, DMSO) *δ:* 0.56–0.67 (m, 2H, CH_2), 0.68–0.77 (m, 2H, CH_2), 2.83–2.95 (m, 1H, CH), 7.34–7.50 (m, 3H, arom H), 7.53–7.66 (m, 3H, arom H), 7.85 (d, *J* = 7.1 Hz, 1H,

arom H), 7.94 (d, *J* = 7.2 Hz, 1H, arom H), 8.01 (s, 1H, arom H), 8.19 (s, 1H, arom H), 8.35 (s, 1H, arom H), 8.59 (bs, 1H, CONH), 8.69 (s, 1H, arom H), 12.30 (bs, 1H, NH) ppm. ^{13}C NMR (75 MHz, DMSO) δ: 6.2 (CH_2), 23.6 (CH), 83.7 (C), 91.2 (C), 95.8 (C), 120.7 (C), 123.7 (C), 125.8 (CH), 126.0 (CH), 126.7 (CH), 128.5 (CH), 129.1 (CH), 129.2 (C), 129.5 (CH), 130.2 (CH), 131.5 (CH), 131.9 (CH), 135.6 (C), 138.9 (C), 143.6 (CH), 148.0 (C), 167.8 (C) ppm. HR-MS m/z $[M+H]^+$ calcd for $C_{29}H_{23}N_3O_2$ 378.1601, found 378.1597.

***N*-Cyclopropyl-3-(3-(thiophen-3-ylethynyl)-1*H*-pyrrolo[2,3-*b*]pyridin-5-yl)benzamide (9e)**. This compound was obtained using the precursor **3g** and 3-(*N*-cyclopropylaminocarbonyl)phenylboronic acid pinacol ester. The crude residue was purified by flash chromatography using a mixture of hexane and acetone (in a ratio of 7:3) as mobile phase, affording the title compound as a beige solid in 39% yield (24.7 mg, 0.06 mmol). ^{1}H NMR (300 MHz, DMSO) δ: 0.57–0.65 (m, 2H, CH_2), 0.68–0.77 (m, 2H, CH_2), 2.81–2.95 (m, 1H, CH), 7.32 (d, *J* = 4.7 Hz, 1H, arom H), 7.57 (t, *J* = 7.6 Hz, 1H, arom H), 7.62–7.68 (m, 1H, arom H), 7.80–7.89 (m, 2H, arom H), 7.93 (d, *J* = 7.6 Hz, 1H, arom H), 7.97 (s, 1H, arom H), 8.18 (s, 1H, arom H), 8.31 (s, 1H, arom H), 8.59 (d, *J* = 2.7 Hz, 1H, CONH), 8.68 (s, 1H, arom H), 12.27 (s, 1H, NH) ppm. ^{13}C NMR (75 MHz, DMSO) δ: 6.2 (CH_2), 23.6 (CH), 82.8 (C), 86.5 (C), 95.9 (C), 120.6 (C), 122.6 (C), 125.8 (CH), 126.0 (CH), 126.7 (CH), 127.1 (CH), 129.2 (C and CH), 129.5 (CH), 130.2 (CH), 131.7 (CH), 135.6 (C), 138.9 (C), 143.6 (CH), 147.9 (C), 167.8 (C) ppm. HR-MS m/z $[M+H]^+$ calcd for $C_{23}H_{17}N_3OS$ 384.1165, found 384.1157.

3-(3-((3-Chlorophenyl)ethynyl)-1*H*-pyrrolo[2,3-*b*]pyridin-5-yl)-*N*-cyclopropylbenzamide (9f). This compound was obtained using the precursor **3e** and 3-(*N*-cyclopropylaminocarbonyl)phenylboronic acid pinacol ester. The crude residue was purified by flash chromatography using a mixture of hexane and acetone (in a ratio of 7:3) as mobile phase, affording the title compound as a white solid in 61% yield (37.8 mg, 0.09 mmol). ^{1}H NMR (300 MHz, DMSO) δ: 0.58–0.66 (m, 2H, CH_2), 0.68–0.77 (m, 2H, CH_2), 2.89 (dt, *J* = 7.0, 3.8 Hz, 1H, CH), 7.42–7.48 (m, 2H, arom H), 7.53–7.62 (m, 2H, arom H), 7.72 (s, 1H, arom H), 7.85 (d, *J* = 7.7 Hz, 1H, arom H), 7.95 (d, *J* = 7.7 Hz, 1H), 8.03 (s, 1H, arom H), 8.19 (s, 1H, arom H), 8.42 (d, *J* = 1.7 Hz, 1H, arom H), 8.59 (d, *J* = 3.8 Hz, 1H, CONH), 8.70 (d, *J* = 1.7 Hz, 1H, arom H), 12.36 (s, 1H, NH) ppm. ^{13}C NMR (75 MHz, DMSO) δ: 6.2 (CH_2), 23.6 (CH), 85.4 (C), 89.9 (C), 95.4 (C), 120.7 (C), 125.8 (C), 126.0 (CH), 126.7 (CH), 128.5 (CH), 129.3 (C), 129.5 (CH), 130.0 (CH), 130.3 (CH), 130.9 (CH), 131.0 (CH), 132.4 (CH), 133.7

(C), 135.6 (C), 138.8 (C), 143.7 (CH), 148.0 (C), 167.8 (C) ppm. HR-MS m/z $[M+H]^+$ calcd for $C_{25}H_{18}ClN_3O$ 412.1211, found 412.1201.

***N*-Cyclopropyl-3-(3-((3-methoxyphenyl)ethynyl)-1*H*-pyrrolo[2,3-*b*]pyridin-5-yl)benzamide (9g)**. This compound was obtained using the precursor **3f** and 3-(*N*-cyclopropylaminocarbonyl)phenylboronic acid pinacol ester. The crude residue was purified by flash chromatography using a mixture of hexane and acetone (in a ratio of 7:3) as mobile phase, affording the title compound as a white solid in 75% yield (46.7 mg, 0.11 mmol). ^1H NMR (300 MHz, DMSO) *δ:* 0.58–0.66 (m, 2H, CH_2), 0.68–0.77 (m, 2H, CH_2), 2.89 (dt, *J* = 7.1, 3.7 Hz, 1H, CH), 3.81 (s, 3H, OCH_3), 6.97 (dd, *J* = 8.2, 1.9 Hz, 1H, arom H), 7.14–7.23 (m, 2H, arom H), 7.34 (t, *J* = 7.9 Hz, 1H, arom H), 7.58 (t, *J* = 7.7 Hz, 1H, arom H), 7.85 (d, *J* = 7.7 Hz, 1H, arom H), 7.95 (d, *J* = 7.7 Hz, 1H, arom H), 8.01 (s, 1H, arom H), 8.20 (s, 1H, arom H), 8.37 (d, *J* = 1.9 Hz, 1H, arom H), 8.59 (d, *J* = 3.7 Hz, 1H, CONH), 8.70 (d, *J* = 1.8 Hz, 1H, arom H), 12.30 (s, 1H, NH) ppm. ^{13}C NMR (75 MHz, DMSO) *δ:* 6.2 (CH_2), 23.6 (CH), 55.7 (OCH_3), 83.6 (C), 91.2 (C), 95.8 (C), 115.0 (CH), 116.3 (CH), 120.7 (C), 124.0 (CH), 124.8 (C), 125.9 (CH), 126.0 (CH), 126.7 (CH), 129.2 (C), 129.5 (CH), 130.3 (CH), 132.0 (CH), 135.6 (C), 138.9 (C), 143.6 (CH), 148.0 (C), 159.7 (C), 167.8 (C) ppm. HR-MS m/z $[M+H]^+$ calcd for $C_{26}H_{21}N_3O_2$ 408.1706, found 408.1694.

Table S1. Anti-VEEV activity and cytotoxicity of 5-aryl-3-(pyridin-3-ylethynyl)- pyrrolo[2,3-*b*]pyridines

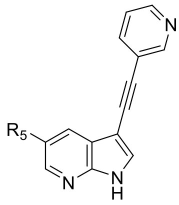

Cmpd#	R_5	VEEV EC_{50} (μM)[a]	U-87 MG CC_{50} (μM)[b]
**1**	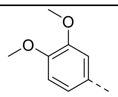	1.22 ± 0.22	>10
**4a**	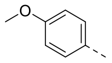	6.81 ± 1.87	>10
**4b**	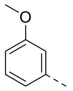	>10	>10
**4c**	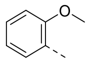	5.34 ± 1.87	>10
**4d**	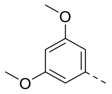	>10	>10
**4e**	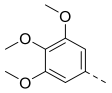	1.50 ± 0.17	>10
**4f**	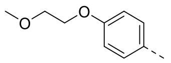	>10	>10
**4g**	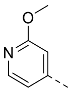	>10	>10
**4h**	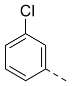	>10	>10
**4i**	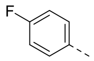	9.05 ± 1.03	>10
**4j**	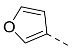	>10	>10
**4k**	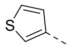	>10	>10

[a]50% effective concentration, or concentration required to inhibit virus-induced cytopathogenicity in U-87 MG cells by 50%
[b]50% cytotoxic concentration, or concentration required to reduce viability of U-87 MG cells by 50%

Table S2. Anti-VEEV activity and cytotoxicity of 5-(2,5-dimethoxy)-3-alkynyl-pyrrolo[2,3-*b*]pyridines

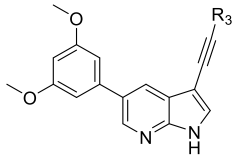

**Cmpd#**	**R_3**	**VEEV EC_{50} (μM)[a]**	**U-87 MG CC_{50} (μM)[b]**
**4d**	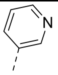	>10	>10
**5a**	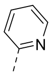	>10	>10
**5b**	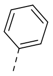	>10	>10
**5c**	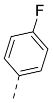	6.27 ± 0.93	>10
**5d**	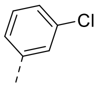	6.13 ± 0.94	>10
**5e**	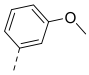	6.29 ± 2.71	>10
**5f**	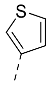	4.30 ± 0.47	>10

[a]50% effective concentration, or concentration required to inhibit virus-induced cytopathogenicity in U-87 MG cells by 50%

[b]50% cytotoxic concentration, or concentration required to reduce viability of U-87 MG cells by 50%

Table S3. Anti-VEEV activity and cytotoxicity of *N*-substituted 5-(2,5-dimethoxy)-3-(3-pyridinyl)-pyrrolo[2,3-*b*]pyridines

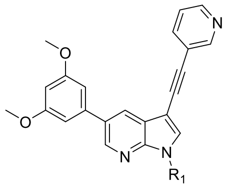

**Cmpd#**	**R$_1$**	**VEEV EC$_{50}$ (μM)^a**	**U-87 MG CC$_{50}$ (μM)^b**
**7a**	methyl	>10	>10
**7b**	ethyl	3.92 ± 1.15	>10
**7c**	isobutyl	>10	>10
**7d**	benzyl	>10	>10

[a]50% effective concentration, or concentration required to inhibit virus-induced cytopathogenicity in U-87 MG cells by 50%
[b]50% cytotoxic concentration, or concentration required to reduce viability of U-87 MG cells by 50%

Table S4. Anti-VEEV activity and cytotoxicity of 5-aryl-3-(3-pyridinyl)pyrrolo[2,3-*b*]pyridines

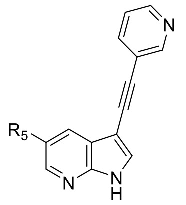

**Cmpd#**	**R$_5$**	**VEEV EC$_{50}$ (μM)[a]**	**U-87 MG CC$_{50}$ (μM)[b]**
**8a**	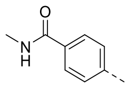	2.72 ± 0.93	>10
**8b**	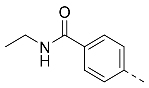	6.01 ± 1.81	>10
**8c**	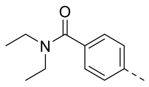	3.49	>10
**8d**	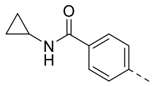	>10	>10
**8e**	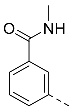	>10	>10
**8f**	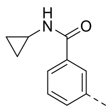	2.75 ± 1.22	>10
**8g**	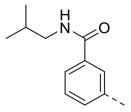	3.75 ± 1.08	>10
**8h**	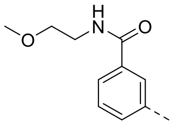	2.48 ± 1.27	>10
**8i**	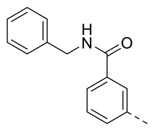	1.29 ± 1.41	>10
**8j**	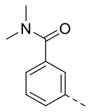	4.57 ± 4.87	>10
**8k**	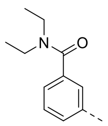	>10	>10

**8l**	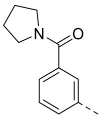	>10	>10
**8m**	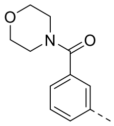	>10	>10

[a]50% effective concentration, or concentration required to inhibit virus-induced cytopathogenicity in U-87 MG cells by 50%
[b]50% cytotoxic concentration, or concentration required to reduce viability of U-87 MG cells by 50%

Table S5. Anti-VEEV activity and cytotoxicity of 5-(*N*-cyclopropylbenzamide)-3-alkynyl-pyrrolo[2,3-*b*]pyridines

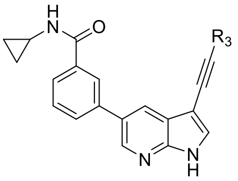

**Cmpd#**	**R_5**	**VEEV EC_{50} (μM)[a]**	**U-87 MG CC_{50} (μM)[b]**
**8f**	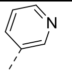	2.75 ± 1.22	>10
**9a**	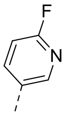	6.54 ± 1.21	>10
**9b**	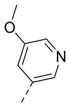	1.39 ± 0.20	8.99 ± 1.60
**9c**	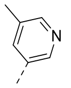	1.80 ± 0.32	>10
**9d**	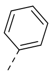	>10	>10
**9e**	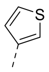	>10	>10
**9f**	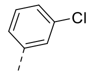	6.70 ± 2.79	>10
**9g**	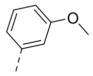	5.27 ± 1.01	>10

[a]50% effective concentration, or concentration required to inhibit virus-induced cytopathogenicity in U-87 MG cells by 50%
[b]50% cytotoxic concentration, or concentration required to reduce viability of U-87 MG cells by 50%
